# Discovery of the Highly Selective and Potent STAT3 Inhibitor for Pancreatic Cancer Treatment

**DOI:** 10.1021/acscentsci.3c01440

**Published:** 2024-02-10

**Authors:** Huang Chen, Aiwu Bian, Wenbo Zhou, Ying Miao, Jiangnan Ye, Jiahui Li, Peng He, Qiansen Zhang, Yue Sun, Zhenliang Sun, Chaowen Ti, Yihua Chen, Zhengfang Yi, Mingyao Liu

**Affiliations:** † Shanghai Key Laboratory of Regulatory Biology, Institute of Biomedical Sciences and School of Life Sciences, East China Normal University, Shanghai 200241, P.R. China; ‡ Shanghai Yuyao Biotech Co., LTD. Shanghai 200241, China; § Southern Medical University Affiliated Fengxian Hospital, Shanghai 201499, China

## Abstract

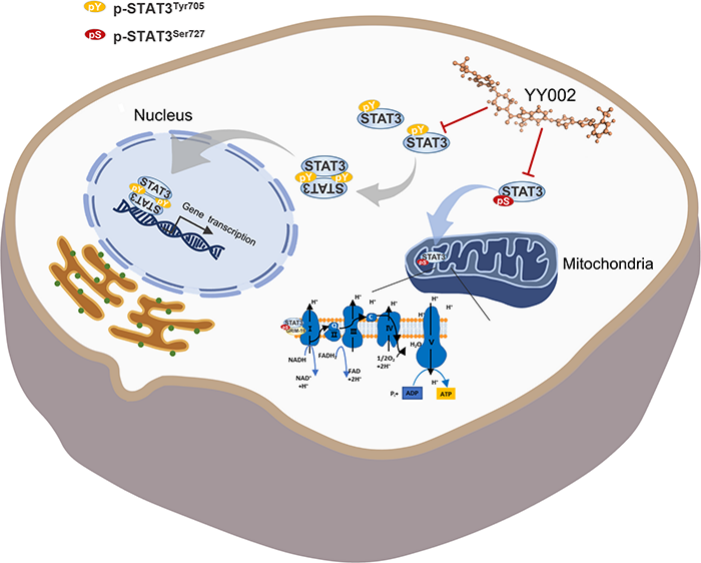

Signal transducer and activator of transcription 3 (STAT3) is an attractive cancer therapeutic target. Unfortunately, targeting STAT3 with small molecules has proven to be very challenging, and for full activation of STAT3, the cooperative phosphorylation of both tyrosine 705 (Tyr705) and serine 727 (Ser727) is needed. Further, a selective inhibitor of STAT3 dual phosphorylation has not been developed. Here, we identified a low nanomolar potency and highly selective small-molecule STAT3 inhibitor that simultaneously inhibits both STAT3 Tyr705 and Ser727 phosphorylation. YY002 potently inhibited STAT3-dependent tumor cell growth *in vitro* and achieved potent suppression of tumor growth and metastasis *in vivo*. More importantly, YY002 exhibited favorable pharmacokinetics, an acceptable safety profile, and superior antitumor efficacy compared to BBI608 (STAT3 inhibitor that has advanced into phase III trials). For the mechanism, YY002 is selectively bound to the STAT3 Src Homology 2 (SH2) domain over other STAT members, which strongly suppressed STAT3 nuclear and mitochondrial functions in STAT3-dependent cells. Collectively, this study suggests the potential of small-molecule STAT3 inhibitors as possible anticancer therapeutic agents.

## Abstract 2

YY002 is the highly selective and potent small molecular inhibitor that simultaneously inhibited STAT3 Tyr705 and Ser727 phosphorylation, demonstrating promising antitumor activity.

## Introduction

Signal transducer and activator of transcription 3 (STAT3) integrates signals from cytokines and growth factors into transcriptional responses in target cells. It mediates a complex spectrum of cellular responses, including proliferation, differentiation, and apoptosis.^1^ STAT3 is a key element in multiple oncogenic signaling pathways, which activate STAT3 by canonical and noncanonical pathways.^2^ The canonical pathway of STAT3 is activated by both receptor tyrosine kinases and nonreceptor tyrosine kinases upon stimulation with various growth factors or cytokines. The activated receptors phosphorylate the Tyrosine705 (Tyr705) residue of STAT3, which leads to STAT3 homodimerization, nuclear translocation and transcriptional activation. It was originally proposed that the reduction of STAT3 Tyr705 phosphorylation would prevent STAT3 homodimerization and consequently inhibit the activation of its target genes. Thus, the majority of drug development efforts have been focused on STAT3-Tyr705 phosphorylation inhibition. However, these inhibitors have limited antitumor activities.^3,4^

Apart from the well-studied Tyr705 activation (canonical pathway), STAT3 undergoes alternative post-translational modifications like phosphorylation of Serine 727 (Ser727),^2^ which mediates STAT3 translocating into mitochondria, and subsequent binding to the electron transport chain to regulate mitochondrial respiration and oxidative phosphorylation (OXPHOS)^5,6^ The Warburg effect describes the phenomenon that tumors are highly reliant on glycolysis for generating the intermediate metabolites to meet the demands of the tumors’ high rate of proliferation.^7^ The recent research also demonstrated that many tumors employ high levels of OXPHOS for energy metabolism and tumor survival.^8−10^ OXPHOS is an emerging drug development target in refractory cancers such as acute myeloid leukemia (AML),^11,12^ pancreatic cancer,^13,14^*SMARCA4* mutant tumors,^15^ and so forth. Some inhibitors target OXPHOS by directly interfering with the electron transport chain (ETC) complex. Among them, IACS-010759^16^ and metformin,^17^ advanced to clinic trials in several advanced tumors. However, directly targeting the ETC has some unexpected toxicities in the clinic.^18^ Thus, the inhibition of STAT3 Ser727 phosphorylation, in turn indirectly inhibiting OXPHOS may provide an alternative approach to achieve metabolic synthetic lethality in cancer cells. In conclusion, the phosphorylation of STAT3 at Tyr705 along with Ser727 is required for full activation of STAT3. Developing a STAT3 inhibitor that simultaneously inhibits STAT3 Tyr705 and Ser727 phosphorylation would achieve an adequate inhibition of STAT3, and may achieve a highly potent therapeutic benefit for cancer treatment.

Pancreatic cancer is an aggressive malignancy with a 5-year survival rate of less than 9%,^19^ which is hard to diagnose and has high liver and lymph node metastasis rates.^20^ Up to date, the therapies of advanced pancreatic cancer are extremely lacking, and the current approved drugs are with limited clinical benefits.^21,22^ Therefore, it is urgent to search, identify and develop novel targeted drug candidates for advanced pancreatic cancer. Overexpression and persistent activation of STAT3 is tightly relevant to the poor prognosis of pancreatic cancer.^23−25^ STAT3 is consistently activated in pancreatic cancer in multiple ways. For instance, myeloid cells produce IL-6 to activate the JAK-STAT3 pathway that promotes the development of pancreatic ductal adenocarcinoma (PDAC) in a Kras^G12D^-driven pancreatic tumor mouse model.^24,26^ Besides, pancreatic-specific Kras and P53 mutations induce an accumulation of reactive oxygen species (ROS) in mouse pancreatic tumors, leading to activation of STAT3. Moreover, genetic approaches to eliminate STAT3 expression or administration of inhibitors of JAK2 or STAT3 contribute to decreasing tumor fibrosis and pancreatic stellate cells and altering the type of immune cells infiltrating the tumor.^27^

In this study, we used two functional assays, the STAT3-Luciferase reporter assay (based on pTyr705 nuclear transcriptional function) and the mitochondrial respiration assay (based on pSer727 mitochondrial OXPHOS function), to screen inhibitors that abolished STAT3 pTyr705 and pSer727 concurrently. After several rounds of screening and rational drug modifications, we identified a series of small-molecule STAT3 inhibitors. The most promising lead, YY002, a highly potent and selective STAT3 inhibitor, inhibited STAT3 Tyr705 and Ser727 phosphorylation, thereby abrogating the STAT3 nuclear and mitochondrial functions. YY002 potently inhibited pancreatic cancer growth and metastasis *in vitro* and *in vivo*. Taken together, this work not only revealed a novel STAT3 Tyr705 and Ser727 phosphorylation inhibitor as a promising new therapeutic agent for pancreatic cancer, but also provided insights into approaches to the development of STAT3 Tyr705 and Ser727 phosphorylation inhibitors.

## Material and Methods

### Chemical, Cell Lines, Culture and Reagents

Detailed information on chemicals and cell lines is described in Supporting Information and Methods. All cell lines were maintained in incubators containing 5% CO_2_ at 37 °C. The AQueous One Solution Cell Proliferation Kit was purchased from Promega (MTS reagent, Madison, WI).

### STAT3 Knockdown

The shRNAs for silencing STAT3 are listed in Supplementary Table S1. ShRNAs were respectively inserted into pLKO.1 vector, then were cotransfected into 293T cells with the packing plasmids pMD2G and pXPAX2, using Lipofectamine 2000 (Thermo Fisher Scientific, Beijing, China). After transfection for 72 h, the harvested lentiviruses were infected into tumor cell lines and puromycin was added to screen for stable STAT3 knockdown cells. Finally, the knockdown efficiency was detected by Western blot.

### Plasmid and Protein Expression

STAT1 (amino acid residues 127–716), STAT2 (amino acid residues 138–702), STAT3 (amino acid residues 127–722), STAT4 (amino acid residues 133–705), STAT5B (amino acid residues 136–703) and STAT6 (amino acid residues 113–658) were cloned into the pET28A vector. STAT3-SH2 (amino acid residues 586–685) and the mutants of the STAT3-SH2 produced by site-directed mutagenesis were also cloned into the pET28A vector. All recombinant human fusion proteins were expressed in *E. coli* BL21 (DE3). Then, according to different induction conditions, the protein expression was induced. During the induction of the expression of STAT3-SH2 and all the mutants, STAT1, STAT2, STAT3 and STAT6, when the OD value reached about 0.6–1.0, the *E. coli* was transferred to 22 °C and 0.5 mM Isopropyl β-D-1-thiogalactopyranoside (IPTG) was added. For the expression of STAT4 and STAT5B when the OD value reached about 0.5–0.6, the *E. coli* was transferred to 30 °C for 2.5–3 h and 0.2 mM IPTG was added.

### Docking Assay

Docking was performed by Schrödinger Glide software (New York, NY, USA). The induced fit docking (IFD) protocol was performed on compound YY002 using the induced fit tool in Maestro 11.5 from Schrödinger. IFD employed the use of Glide and Prime for ligand docking and protein refinement, respectively. The active site was centered on the Tyr705 phosphorylated site near the STAT3-SH2 domain (PDB code 6QHD. Deposited: 2019-01-16; Released: 2019-06-19). Induced fit protein–ligand complexes were generated using Prime and further subjected to side chain and backbone refinement.

### Microscale Thermophoresis (MST) Assay

Microscale thermophoresis (MST) was conducted as previously described.^28^ Binding affinities of YY002, Stattic against purified STAT3^127–722^ and STAT3-SH2 protein or YY002 against STAT3-SH2 WT and mutants were measured by a Monolith NT.115 (Nanotemper Technologies). The proteins were fluorescently labeled according to the manufacturer’s procedure and kept in the MST buffer (50 mM HEPES, pH 7.0, 500 mM NaCl, 0.01% NP40, 50 mM l-argine) at a concentration of 200 nM. Next, the RED fluorescent dye was added, mixed and incubated for 30 min at 25 °C in the dark. For each assay, the labeled protein was mixed with the same volume of unlabeled compound at 16 different serially diluted concentrations at room temperature. The samples were then loaded into premium capillaries (NanoTemper Technologies) and measured at 25 °C using 20%–40% LED power and medium MST power. Each assay was repeated two or three times. Data analyses were performed using MO. Affinity Analysis v.2.2.4 software. All figures were made by GraphPad Prism 7.0.

### Surface Plasmon Resonance (SPR) Assay

Surface plasmon resonance (SPR) experiments were performed with a Biacore 8K instrument (Cytiva) with CM5 sensor chip (Cytiva). To test YY002 binding of STAT3 protein, serially diluted concentrations of YY002 were injected into the flow system. Experiments were conducted using the buffer: 10 mM PBS, pH7.4, 137 mM NaCl, 2.7 mM KCl, 1 mM DTT, 0.05% P20, 5% DMSO. Recombinant full-length human STAT3 (Creative Biomart, Catalog: STAT3–001H, Batch number: PSS1072207) was immobilized on the sensor chip (CM5) using the amine-coupling method according to standard protocols. The analyte was injected at the flow rate of 30 μL/min. The association time was 180 s and the dissociation time was 180 s. Since YY002 was dissolved in PBS containing 5% dimethyl sulfoxide and a solvent correction assay was performed to adjust the results. YY002 at various concentrations was injected into the flow system. The *K*_D_ values were calculated with the kinetics and affinity analysis option of Biacore evaluation software.

### Colony Formation

Tumor cells were seeded into 6-well plates, allowing attachment overnight. The test compounds were added at indicated concentrations for 1 week of treatment. Then colonies were fixed by 4% paraformaldehyde (PFA) and then stained with 0.2% crystal violet. Images were photographed using a digital camera, and colonies were quantified by manual counting.

### Western Blot

Pancreatic cancer cells were lysed in RIPA buffer supplemented with 1 mM phenylmethylsulfonyl fluoride (PMSF), a proteinase inhibitor cocktail and a phosphatase inhibitor cocktail (Sigma). Lysates were separated by 8–12% sodium dodecyl sulfate-polyacrylamide gel electrophoresis (SDS-PAGE) and transferred to nitrocellulose. The blots were probed with specific antibodies followed by a secondary antibody, and then membranes were examined by the LI-COR Odyssey infrared imaging system (LI-COR Biotechnology, Lincoln NE). The antibodies used and their technical information are listed in Supplementary Table S2.

### Real-Time Quantitative PCR (RT-qPCR)

Total RNA was isolated using TRIzol reagent (Takara, Japan). RNA was used to generate single-stranded cDNA according to the manufacturer’s protocols of the cDNA Reverse Transcription Kit (Takara, Japan) and then subjected to polymerase chain reaction (PCR) using SYBR Green Master Mix (Thermo Fisher Scientific). The expression of VEGF, Cyclin D1, c-Myc and Bcl-2 in Capan-2, HPAC, and BxPC-3 cells was validated by real-time quantitative PCR. The sequences of Real-time PCR primers are listed in Supplementary Table S3.

### Luciferase Reporter Assay

The STAT3 luciferase plasmids (pGL4.47 luc2p/SIE/Hygro) vector (E4041, Promega) and Renilla plasmids were cotransfected into HEK293T with Lipofectamine 2000 reagent (Invitrogen) and incubated for 20–24 h. Then, the cells were seeded into 96-well plates at a cell density of 2 × 10^4^ per well. After overnight attachment of the cells, compounds were added with concentration gradient, and IL-6 (20 ng/mL) was used as an activator of STAT3. Luciferase assays were performed by using the Dual Luciferase Reporter Assay System (Promega) according to manufacturer’s instructions. The firefly luciferase activities were normalized against Renilla luciferase activities.

### Immunofluorescence Assay

BxPC-3 and Capan-2 cells were cultured on glass coverslips at a density of 10^5^ per well of 24-well plate. After treating with YY002 for 24 h, IL-6 was added for 30 min stimulation. Then, the culture medium was removed, and the cell slides were fixed with 4% paraformaldehyde for 15 min and permeated by 0.2%Triton X-100 for 20 min. Next, after PBS wash and blocking in 0.5% BSA, primary antibodies were added to incubate with cells overnight at 4 °C, and then, cells were incubated with corresponding secondary antibodies for 1 h at room temperature. Finally, DAPI was added and a confocal microscope (Leica) was used to record images.

### Mitochondrial Bioenergetics Analysis

Mitochondrial bioenergetics were evaluated by oxygen consumption rate (OCR) and extracellular acidification rate (ECAR), using the Seahorse XFe96 analyzer (Agilent Technologies). OCR reflected oxidative phosphorylation, and during the experiment, 1 × 10^4^ to 2 × 10^4^ cells per well were seeded onto the cell culture plates in the kit. After treatment with YY002 for 1 h, the medium in the plates was replaced by the newly configured detection medium through the liquid exchange program of the Seahorse instrument. Then, following basal respiration, the mitochondrial effectors (oligomycin, FCCP, and a mixture of rotenone and antimycin A) were injected sequentially according to the manufacturer’s protocol to measure the ATP-linked respiration and spare respiratory capacity. During the process of ECAR (which reflected glycolysis), the density of cells per well and the time of YY002 treatment were consistent with experiments to measure OCR. Glucose, oligomycin and 2-DG were injected sequentially to evaluate the level of cellular glycolysis and the ability of cells to restore glycolysis.

### Animal Studies

The mice were obtained from the Animal Center of East China Normal University. All animal experiments conformed to the Animal Investigation Committee of the Institute of Biomedical Sciences, East China Normal University. NOD/SCID, male, 6–8-week-old mice were injected subcutaneously with 5 × 10^6^ PANC-1 and MIA Paca-2 cells in a mixture of PBS with 50% Matrigel for tumor growth. Female Balb/c Nude was inoculated with 1 × 10^7^ MDA-MB-231 tumor cells (in 0.15 mL, 1:1 with Matrigel) at the right flank. Male CB17 SCID was inoculated with 3 × 10^6^ SUDHL-1 tumor cells (in 0.1 mL, 1:1 with Matrigel) at the right flank. Tumor-bearing mice was randomly assigned to different experimental groups for experiments after the tumor nodules grew to 100–200 mm^3^ and the dose formulation of YY002 was as indicated. The tumor volume and mouse body weight were measured every 4–5 days. The tumor volume was calculated using the following equation: tumor volume (*V*) = length × width × width × 0.52. In each group, the relative tumor volume (RTV) was calculated as RTV = *V*_t_/*V*_0_ (*V*_t_ represents the tumor volume at the end of the experiment, V_0_ represents the tumor volume at the beginning of the treatment). The tumor growth inhibition (TGI) was calculated using the following formula: TGI = (1 – RTV_the treated group_ /RTV _the control group)_ × 100%. At the indicated time-points, mice were euthanized and tissue samples were collected for analyses.

### Orthotopic Pancreatic Cancer Tumor Model

Murine pancreatic cancer cells, PAN02-luciferase, suspended into the precooled Matrigel (BD biosciences) were injected (5 × 10^5^ cells per mouse) into the pancreas of male, 6–8-week-old, C57BL/6 mice. The fluorescence was detected 1 week after tumor cell implantation by an IVIS Imaging System (Xenogen Corporation, Alameda, CA) and the mice were divided into 4 groups according to the fluorescence value, and keep the average luminescence values of each group consistent when mice were grouped. The average luminescence values of each group when grouped were as follows: 307714.29 photon/sec^2^/cm^2^/sr (Control), 301142.86 photon/sec^2^/cm^2^/sr (5 mg/kg), 302500 photon/sec^2^/cm^2^/sr (10 mg/kg), 303414.29 photon/sec^2^/cm^2^/sr (10 mg/kg). The dose formulation of YY002 was as indicated. Then, the bioluminescence of cancer cells and body weight of mice were observed once a week during the treatment by IVIS. In addition, we used AsPC-1 cells to construct another orthotopic model of pancreatic cancer (similar to PAN02-Luci), and 2 × 10^6^ cells were injected into male, 6–8-week-old Balb/c mouse. Another independent animal experiment was performed to determine whether YY002 prolongs the survival of mice.

### Liver Metastasis Model of Human Pancreatic Cancer

Murine pancreatic cancer cells, PAN02-luc, suspended into the precooled Matrigel (BD Biosciences) were injected (5 × 10^5^ cells per mouse) into the spleen of male, 6–8-week-old, C57BL/6 mice. The fluorescence was detected after tumor cell implantation for 1 week by an IVIS Imaging System (Xenogen Corporation, Alameda, CA), and the mice were divided into 4 groups according to the fluorescence value. The average luminescence values of each group were kept consistent when mice were grouped. The average luminescence values of each group when grouped were as follows: 45328.57 photon/sec^2^/cm^2^/sr (Control), 44522.29 photon/sec^2^/cm^2^/sr (5 mg/kg), 45885.71 photon/sec^2^/cm^2^/sr (10 mg/kg), 45742.86 photon/sec^2^/cm^2^/sr (10 mg/kg). The dose formulation of YY002 was as indicated. Then, the bioluminescence and body weight of mice were observed once a week during the treatment by IVIS. Another independent animal experiment was performed to determine whether YY002 prolongs the survival of mice.

### Statistical Analysis

Experiments were carried out with three or more replicates. Statistical analyses were done by Student’s *t* test. *P* values < 0.05 were considered significant. The differences between control and experimental groups were determined by one-way ANOVA. Since treatment and time course were investigated, two-way ANOVA followed by post hoc test was also applied. Data were expressed as means and 95% confidence intervals and *P* < 0.05 was considered significant. All analyses were performed using Microsoft Excel 2010 and GraphPad Prism 7 software.

## Results

### Development of Highly Potent and Selective STAT3 Inhibitors

**Figure 1:**
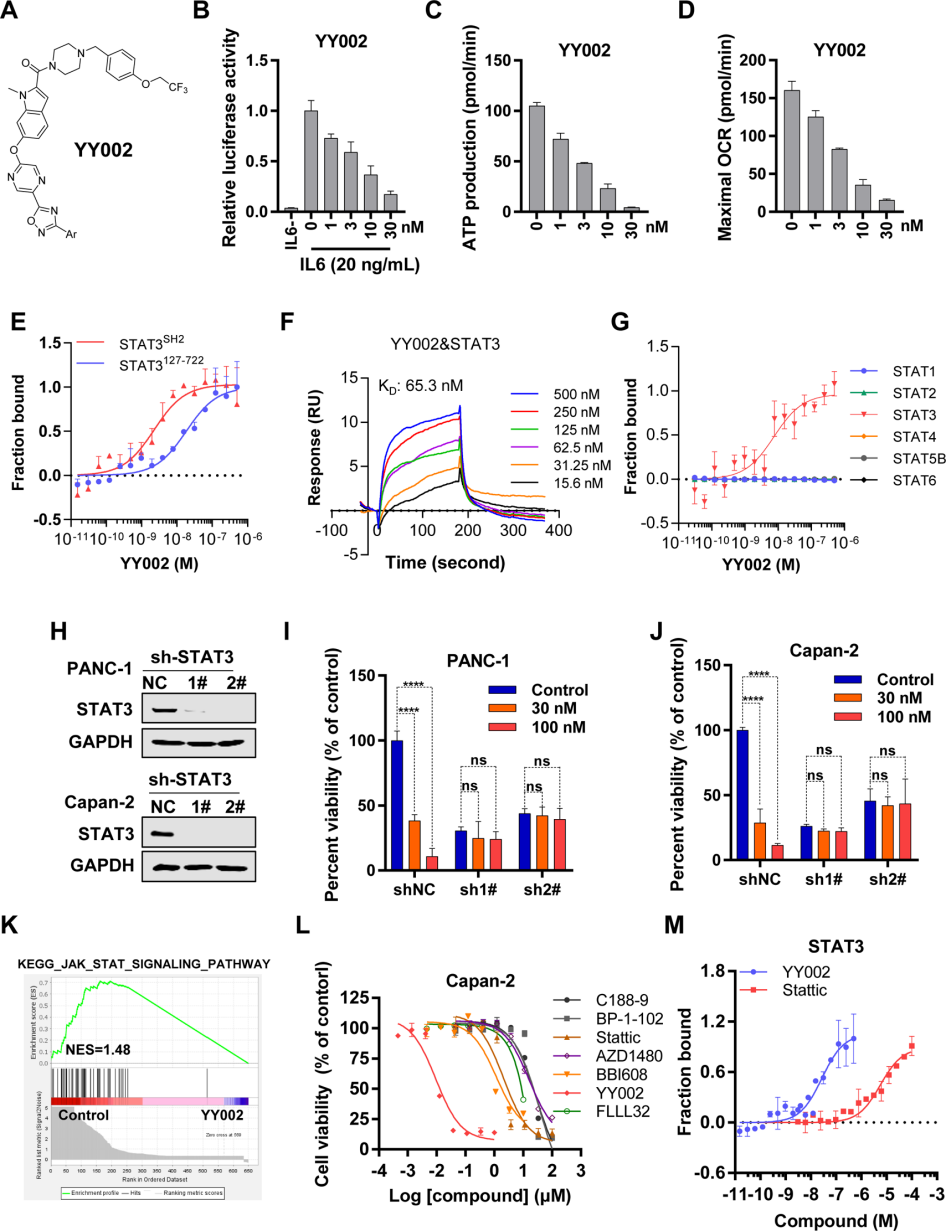
YY002 selectively targets STAT3. (A) Structure of YY002. (B) YY002 inhibited the STAT3 luciferase reporter activity. IL-6 was used as an activator of STAT3 (*n* = 2). (C) YY002 inhibited the ATP production (*n* = 2). (D) YY002 inhibited the OXPHOS rate (OCR = oxygen consumption rate, *n* = 2). (E) Direct binding of YY002 to STAT3^SH2^ or STAT3^127–722^ was determined by MST experiments (*n* = 3). (F) The binding affinities between YY002 and STAT3 was detected by surface plasmon resonance (SPR) assay. (G) YY002 selectively bound to STAT3 and other STAT members (*n* = 3). (H) The shRNA knock-down efficiency in PANC-1 and Capan-2 was confirmed by Western Blot. (I, J) The pancreatic cancer cell lines shNC, shSTAT3-1# and shSTAT3-2# of PANC-1 (I) and Capan-2 (J) were treated with YY002, and the proliferation was determined after 72 h of treatment (*n* = 3). (K) BxPC3 cells were treated with 50 nM YY002 for 24 h, and then RNA-Seq assay was performed. The results were analyzed by gene set enrichment analysis (GSEA). (L) Capan-2 cells were treated with different concentrations of STAT3 inhibitors or YY002, and the proliferation was tested by MTS (*n* = 2). (M) YY002 and Stattic directly bound to STAT3^127–722^, The binding affinities were determined by MST experiments (*n* = 3). Data shown as mean ± SD ns, *P* > 0.05, *P < 0.05, **P < 0.01, *** *P* < 0.001 and **** *P* < 0.0001 by one-way ANOVA following multiple comparison.

To specifically identify STAT3 inhibitors, structure-based virtual screening and computer-aided drug design were utilized. And then, we generated a STAT3-specific luciferase reporter system to confirm compounds capable of inhibiting STAT3 Tyr705 and Ser727 phosphorylation (Supplementary Figure S1A–C) and a mitochondrial respiration assay (Supplementary Figure S1D, S1E). Through these preliminary assays, the small molecule YY002 (Figure 1A, Supplementary Figure S1) exhibited the best inhibitory activity among the investigated compounds. YY002 inhibited STAT3 luciferase reporter expression (Figure 1B), ATP production (Figure 1C) and OXPHOS (Figure 1D) in a concentration-dependent manner, with an inhibitory concentration (IC_50_) ranging from 1 to 10 nM. We next purified the STAT3-SH2 domain (SH2D) and STAT3^127–722^ to confirm whether YY002 directly interacted with STAT3. In the Microscale Thermophoresis (MST) assay, YY002 directly bound STAT3^SH2^ and STAT3^127–722^ with a *K*_D_ of 2.24 ± 0.55 nM and 18.27 ± 4.04 nM, respectively (Figure 1E). The surface plasmon resonance (SPR) assay also proved that YY002 bound to STAT3 with high affinity (Figure 1F). To further determine whether YY002 selectively bind to STAT3 over STAT family proteins, we purified STAT family proteins and performed direct binding assays between YY002 and STATs protein in MST assays. YY002 exhibited a high selectivity against STAT3 with no significant binding affinities to other STAT family proteins (Figure 1G). To further evaluate the dependency of YY002 action on STAT3, we constructed shRNA-mediated STAT3 knockdown stable cell lines, and in cell viability assays, both lines of shSTAT3 PDAC cells (Capan-2, PANC-1) saw a loss of YY002 treatment efficacy upon STAT3 knockdown (Figure 1H–J). This indicates a general lack of off-target effects of YY002 on cell viability. Subsequently, we identified the JAK-STAT3 signaling pathway as significantly affected upon YY002 treatment (Figure 1K). In brief, these results demonstrated that YY002 selectively suppressed the proliferation of tumor cells by targeting STAT3.

Currently, the majority of STAT3 inhibitors merely inhibited STAT3 Tyr705 phosphorylation, and most of them had poor inhibitory activities. So, we compared the inhibitory activity of a STAT3 dual-phosphorylation inhibitor YY002, with the several representative STAT3 inhibitors including STAT3-SH2D inhibitors (C188-9, BP-1–102, Stattic, FLLL32), JAK2 inhibitor (AZD1480), and STAT3 and cancer stemness inhibitor (BBI-608). YY002 exhibited markedly potent antitumor activity and direct binding affinities than the above STAT3 inhibitors; these activities were approximately 1000-fold better than the tested alternatives (Figure 1L,M, Supplementary Figure S2A).

### YY002 Directly Binds to the STAT3-SH2 Domain

**Figure 2:**
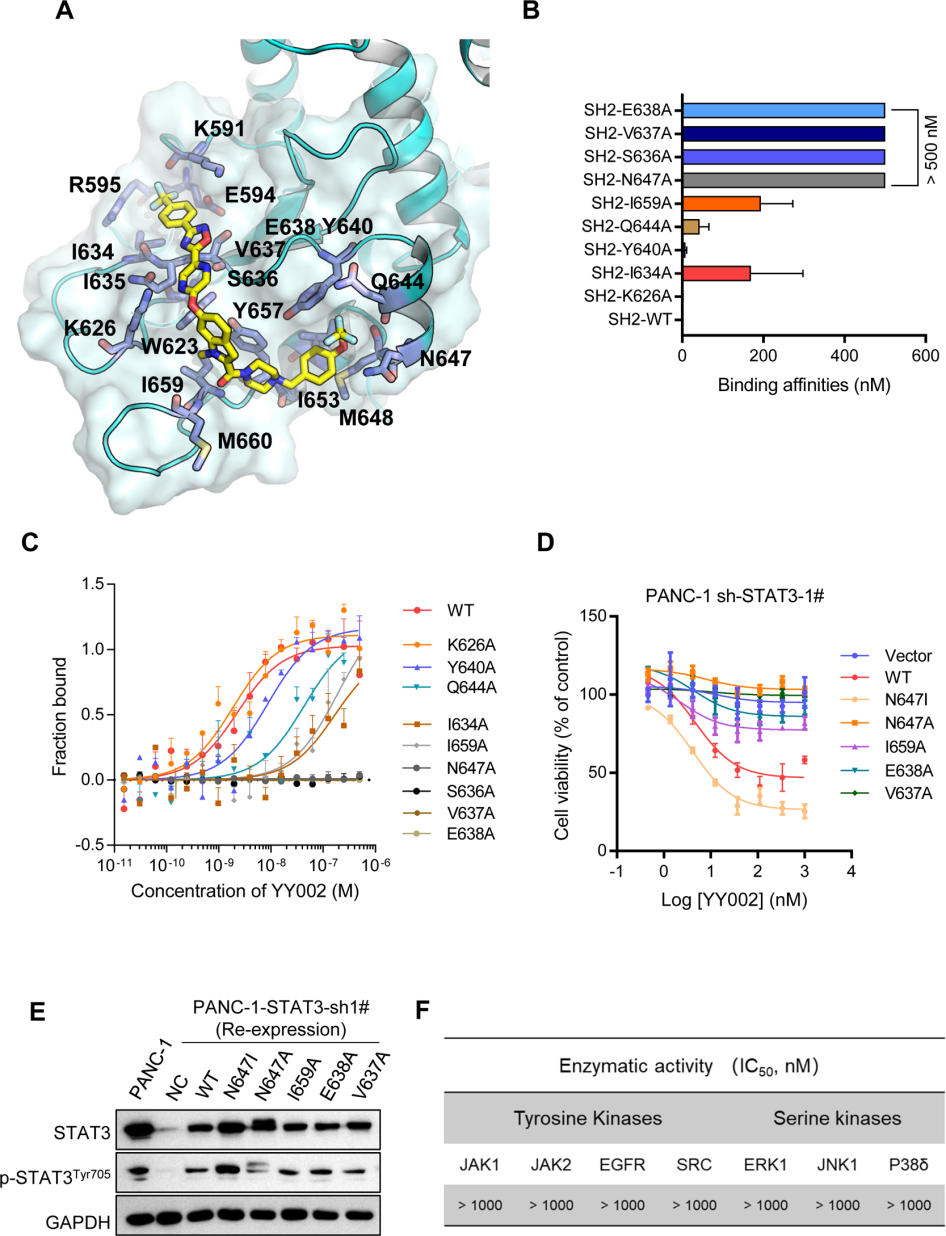
YY002 directly binds to STAT3 SH2 domain. (A) Computer docking model predicting that YY002 bound to the STAT3-SH2 domain. (B, C) YY002 binding to STAT3-SH2 and its indicated mutants. The binding affinities were measured by MST experiments (*n* = 3). (D) PANC-1 shSTAT3-1# cells that were infected with indicated lentivirus vectors to reintroduce specific SH2 mutants were treated with indicated YY002 concentrations, and cell viability was measured (*n* = 3). Data shown as mean ± SD. (E) Re-expression of STAT3 mutant in STAT3 knockdown PDAC cells. The shSTAT3-1# PANC-1 cells that were infected with the indicated lentivirus expression vectors, and the transfection efficacy was detected by Western blots. (F) *In vitro* enzyme inhibition assays.

To illustrate a structural basis for the selective and high binding affinity between YY002 and STAT3, we performed a computational docking and molecular dynamics simulation assay of YY002 and the STAT3 Src homology 2 domain (SH2D), which revealed that the predominant binding mode was within the STAT3-SH2D (Figure 2A). Besides, YY002 had a broader interaction interface with STAT3-SH2D compared with the existing STAT3 inhibitors (Supplementary Figure S2B,C). Specifically, YY002 formed an extensive hydrogen-bonding network with the side chains of the residues of Ser636, Val637, Glu638, Asp647, and Ile659. The residues of Trp623, Ile634, and Gln644 may interact with YY002 through hydrophobic interactions (Figure 2A). Subsequently, we validated the predicted amino acids that YY002 directly bound to by site-directed mutagenesis and determining the binding affinities between the STAT3-SH2D mutants and YY002. As our results showed, the binding affinities between YY002 and the SH2D mutants including K626A, Y640A, and Q644A were only modestly changed compared with the binding affinities of YY002 to SH2D-WT (Figure 2B,C). In contrast, YY002 did not bind several mutants including S636A, V637A, E638A, N647A, I634A and I659A (Figure 2B,C). Thus, computational modeling and direct binding results demonstrated that YY002 directly bound to the residues of S636, V637, E638, N647, I634, and I659. To further confirm whether YY002-binding amino acids were responsible for the anticancer activity of YY002, we overexpressed each mutant in STAT3 stable knock-down PANC-1 cells and treated the above cells with YY002, however, overexpression of the mutants, except for an activated STAT3 mutation N647I^29^ that harbors high pSTAT3, failed to restore the anticancer capacity of YY002 (Figure 2D,E), demonstrating YY002-bound amino acids directly regulated its antipancreatic cancer activity. Furthermore, this led us to investigate if these amino acids also play a crucial role in the function of STAT3. Thus, we performed a STAT3 reporter luciferase assay and found that some mutants such as N647A, I659A, E638A, and V637A failed to respond to IL-6 stimulation of STAT3 transcription (Supplementary Figure S2D), indicating that these amino acid residues are critical for STAT3 transcription.

Taken together, YY002 had a broader interaction interface with STAT3-SH2D, the binding residues and model of YY002 to STAT3-SH2 domain were clarified by computational modeling and direct binding assays. The further results demonstrated that these amino acids were essential for the YY002 binding capabilities and antitumor functions. Moreover, owing to the specific binding between YY002 and STAT3, YY002 also showed no obvious inhibition on tyrosine kinases or serine kinases (Figure 2F).

### YY002 Inhibits Cancer Cell Proliferation, STAT3 Tyr705 Phosphorylation and STAT3 Nuclear Functions

**Figure 3:**
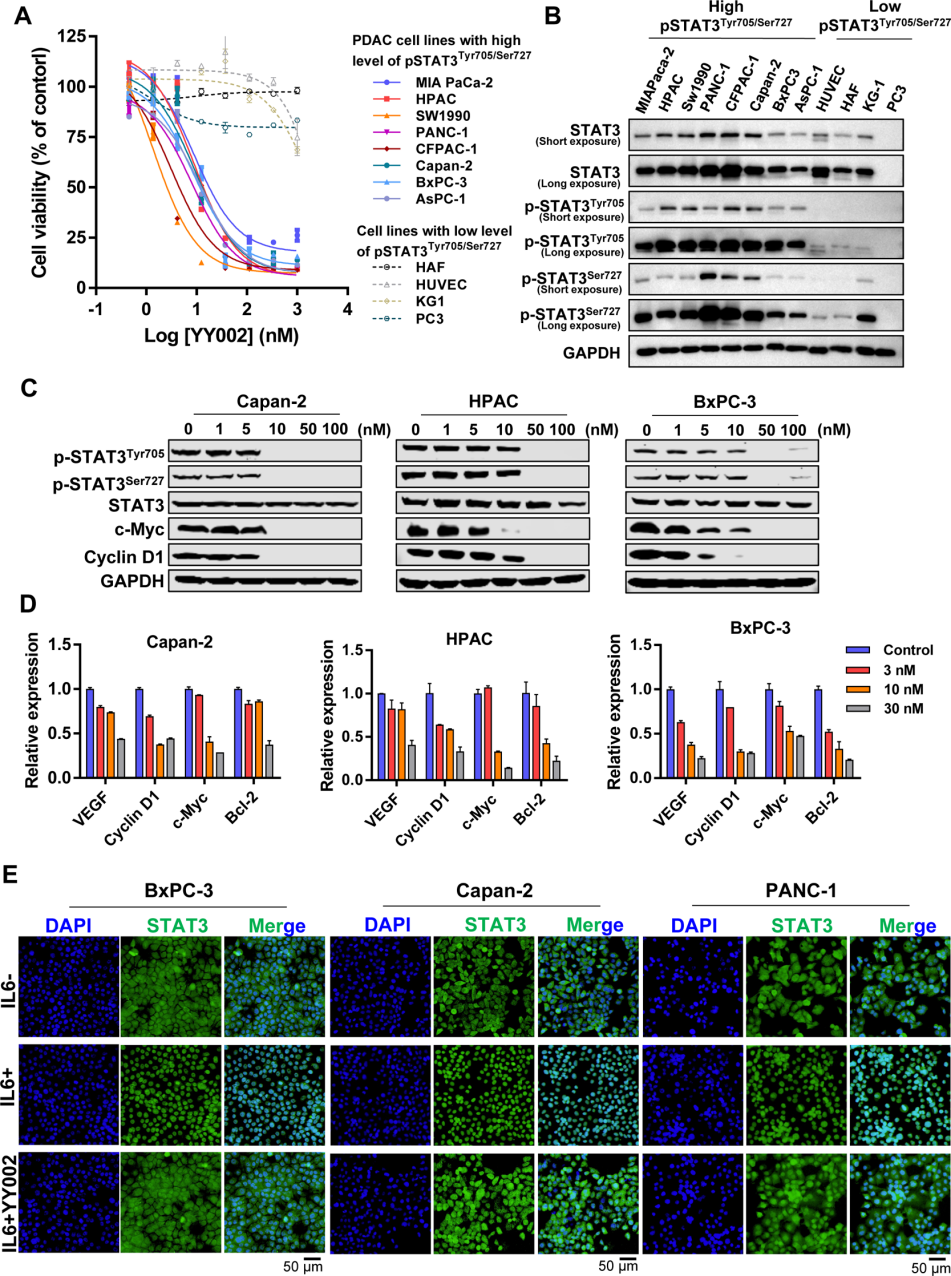
YY002 inhibits pancreatic cancer cell growth, STAT3 Tyr705 phosphorylation and STAT3 nuclear function. (A) YY002 inhibited the proliferation of pancreatic cancer cell lines but only had a limited effect on normal cells (*n* = 2). (B) The protein expression level of STAT3, p-STAT3^Tyr705^ and p-STAT3^Ser727^ in different cell lines. (C) STAT3 phosphorylation and downstream gene expression were measured by Western Blot in Capan-2, HPAC and BxPC-3 cells after treatment with YY002. (D) Down-stream gene expression was measured by quantitative real-time PCR in Capan-2, HPAC and BxPC-3 cells after treatment with YY002 (*n* = 2). (E) YY002 inhibited the entry of STAT3 into the nucleus of BxPC3, Capan-2 and PANC-1 cells. Scale bar, 50 μm. Data shown as mean ± sd. ns, *P* > 0.05, *P < 0.05, **P < 0.01, ***P < 0.001 and ****P < 0.0001 by one-way ANOVA following multiple comparison.

Given that the rapid proliferation of cancer cells is one of the main drivers responsible for tumor progression, we next investigated the antitumor activity of YY002 against several pancreatic cancer cell lines. Because STAT3 is frequently activated in human tumors, it is conceivable that high pSTAT3 (Tyr705 and Ser727) cell lines are more strongly dependent on STAT3 signaling for survival, and that inhibition of STAT3 by YY002 may achieve strong growth inhibitory activity. YY002 potently suppressed pancreatic cancer cells that were with high pSTAT3 (Tyr705 and Ser727), with inhibitory concentrations (IC_50_) ranging from 3 to 11 nM (Figure 3A,B). While, in the low level of p-STAT3 cell lines, including two cancer cell line PC3 and KG1, and normal cell line HUVEC and HAF, YY002 was relative insensitive to these cell lines (Figure 3A,B). Moreover, YY002 inhibited colony formation of multiple STAT3-dependent PDAC cells at concentration of 1 to 10 nM (Supplementary Figure S3A), while YY002 had little impact on STAT3 negative PC3 cells (Supplementary Figure S3B). These results further demonstrated that inhibition of phosphorylated STAT3 (Tyr705 and Ser727) byYY002 is responsible for the anticancer activity of YY002.

We next evaluated the efficacy of YY002 on STAT3 phosphorylation and the expression of its downstream genes on several pancreatic cancer cells. It is widely believed that Tyr705 phosphorylation is closely involved in STAT3 canonical nuclear function, whereas Ser727 phosphorylation regulates mitochondrial electron transfer chain activity. Distinct from most of the reported STAT3 inhibitors, YY002 showed concurrent inhibition of STAT3 Tyr705 and Ser727 phosphorylation (Figure 3C). At the same time, YY002 significantly suppressed STAT3 downstream gene expression in pancreatic cancer cells (Capan-2, HPAC and BxPC-3) (Figure 3C,D). Next, we deduced that YY002 bound to STAT3-SH2D suppressed STAT3 dimerization and reduced STAT3 nuclear translocation. We validated this hypothesis by immunofluorescence experiments. The classical STAT3 stimulatory molecule IL6 promoted STAT3 entry into the nucleus, whereas YY002 treatment significantly inhibited STAT3 nuclear translocation (Figure 3E). In summary, YY002 selectively inhibited pancreatic cancer cell proliferation and suppressed STAT3 Tyr705 and Ser727 phosphorylation. Finally, YY002 inhibited STAT3 classical nuclear function, thus restraining the oncology-related gene expression.

### YY002 Inhibits STAT3 Ser727 Phosphorylation and Mitochondrial OXPHOS

**Figure 4:**
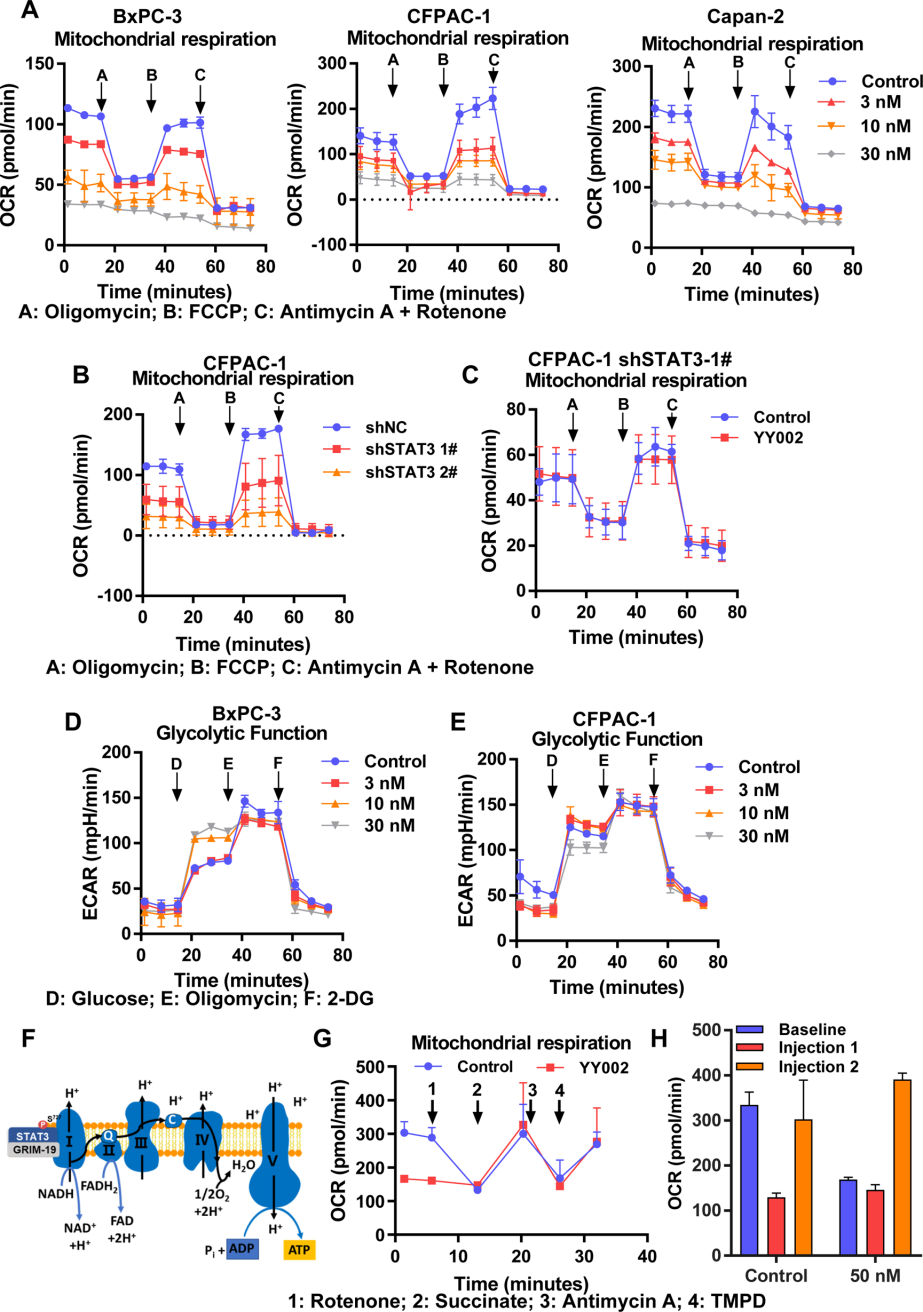
YY002 inhibits STAT3 Ser727 phosphorylation and mitochondrial OXPHOS. (A) Oxygen consumption rate (OCR) was evaluated by the Seahorse XF96 extracellular flux analyzer and OXPHOS was inhibited in BxPC-3 (*n* = 2), CFPAC-1 (*n* = 3) and Capan-2 (*n* = 4) cells treated with YY002. (B) Knockdown of STAT3 in CFPAC-1 inhibited OXPHOS (*n* = 3). (C) CFPAC-1 shSTAT3-1# cells were treated with YY002 or vehicle (DMSO), and the level of OXPHOS was measured (*n* = 3). (D, E) The glycolysis level of BxPC-3 (D) and CFPAC-1 (E) cells treated with different concentrations of YY002 was determined (*n* = 3). (F) A brief description of the mitochondrial electron transport chain. (G, H) Capan-2 cells were treated with 50 nM YY002 or vehicle control and injected with the indicated drugs 1, 2, 3, 4 sequentially, and the mitochondrial respiration was determined by Seahorse instrument assay (*n* = 3). Data shown as mean ± sd.

YY002 significantly inhibited STAT3-Ser727 phosphorylation and mitochondrial STAT3 was prevalently phosphorylated at Ser727, which enhances its mitochondrial functions. We speculated that YY002 would inhibit the STAT3 functions in the mitochondria by suppressing pSTAT3-Ser727. We first investigated the effects of YY002 treatment on pancreatic cancer’s two major energy-generating pathways: mitochondrial oxidative phosphorylation (OXPHOS) and glycolysis. To this end, we measured the oxygen consumption rate (OCR), an indicator of OXPHOS, and the lactate production rate, an indicator of glycolysis (extracellular acidification rate; ECAR). As shown in Figure 4A, YY002 suppressed OXPHOS in pancreatic cancer cells in a concentration-dependent manner.

To confirm whether the above inhibitory effect was due to the direct inhibition of STAT3, STAT3 stable knockdown pancreatic cancer cells were constructed, and the OXPHOS was significantly inhibited upon STAT3 stable knockdown (Figure 4B). Further, we treated STAT3 knockdown cells with YY002, and showed no significant inhibition of OXPHOS (Figure 4C). Subsequently, we reintroduced wild-type STAT3 or the indicated SH2 mutants in stable STAT3 knock-down cells. In WT-STAT3 re-expression cells, YY002 inhibitory capacity was restored, and this effect was specific because it did not fully restore upon expression of the indicated SH2 mutant or vector plasmids (Supplementary Figure S4A, S4B). Moreover, mitochondrial membrane potential (MMP) was also drastically affected in cells treated with YY002 (Supplementary Figure S4C). In contrast, YY002 showed no significant impact on glycolysis in PDAC cells (Figure 4D,E). Taken together, YY002 inhibited STAT3 Ser727 phosphorylation, and then interfered with mitoSTAT3 and its mitochondrial function.

Recent studies showed that STAT3 enters mitochondria and directly binds to electron transport chain complexes to modulate mitochondrial oxidative phosphorylation (Figure 4F). We next confirmed whether YY002 specifically impaired electron transfer chain complexes to inhibit OXPHOS. The electron transfer chain experiments confirmed that treatment of detergent-permeabilized cells with YY002 in medium supplemented with pyruvate and malate (to generate NADH for use by complex I) resulted in an attenuated oxygen consumption rate, whereas when the medium was supplemented with succinic acid to supply complex II, the OCR was not affected by YY002 treatment, thus bypassing the requirement for complex I function, all these results indicated that YY002 selectively impaired complex I functions in PDAC cells (Figure 4G,H). Studies have shown that the inhibition of complex I significantly induces the production of high levels of reactive oxygen species (ROS), leading to oxidative stress.^30^ We used the ROS dye 2′,7′-Dichlorodihydrofluorescein diacetate (DCFH-DA) to further investigate the effect of YY002 on ROS levels. As expected, YY002 significantly increased ROS levels in PDAC cells (Supplementary Figure S4D).

YY002 induction of mitochondrial STAT3 dysfunction as well as suppression of cancer cell OXPHOS suggested that cancer cells may be more sensitive to the cytotoxic effects of YY002 under conditions of increased dependence on mitochondrial activity. Indeed, combination YY002 with an inhibitor of glucose uptake and metabolism, 2-deoxy-d-glucose (2-DG), increased its cytotoxic effects in pancreatic cancer cells (Supplementary Figure S4E,F). Besides, YY002 robustly inhibited PDAC cells growth in galactose-containing medium, wherein cells were rendered dependent on OXPHOS for survival^31^ (Supplementary Figure S4G).

In summary, YY002 targeted STAT3 to inhibit oxidative phosphorylation, but not glycolysis, and further experiments demonstrated that YY002 directly inhibited the function of ETC complex I.

### YY002 Potently Suppresses the Pancreatic Tumor Growth *in Vivo*

To evaluate the antitumor effect of YY002 *in vivo*, we first determined the pharmacokinetic properties of YY002. The plasma exposure and pharmacokinetic parameters were detected following oral and intravenous administration at 10 and 1 mg/kg, respectively. The Cmax of YY002 was 4307.80 ng/mL in mice under oral administration. The *t*_1/2_ value of oral administration of YY002 was 14.30 h. After intravenous administration, YY002 Cmax was 8154.40 ng/mL at a mean *t*_1/2_ of 16.60 h (Supplementary Figure S5A). These results proved that YY002 had an acceptable biological half-life and a moderate time–concentration curve. Importantly, YY002 exhibited well oral bioavailability with a rate of 31.30% in mice (Supplementary Figure S5A).

The drug-like properties of YY002 were also evaluated. For instance, YY002 was stable in human, murine, canine, rat, and monkey plasma, as well as in liver microsomes (Supplementary Figure S5B). In a general safety panel, YY002 showed no obvious inhibition of the human Ether-à-go-go-Related Gene (hERG) and cytochrome P450 enzymes (Supplementary Figure S5C).

**Figure 5:**
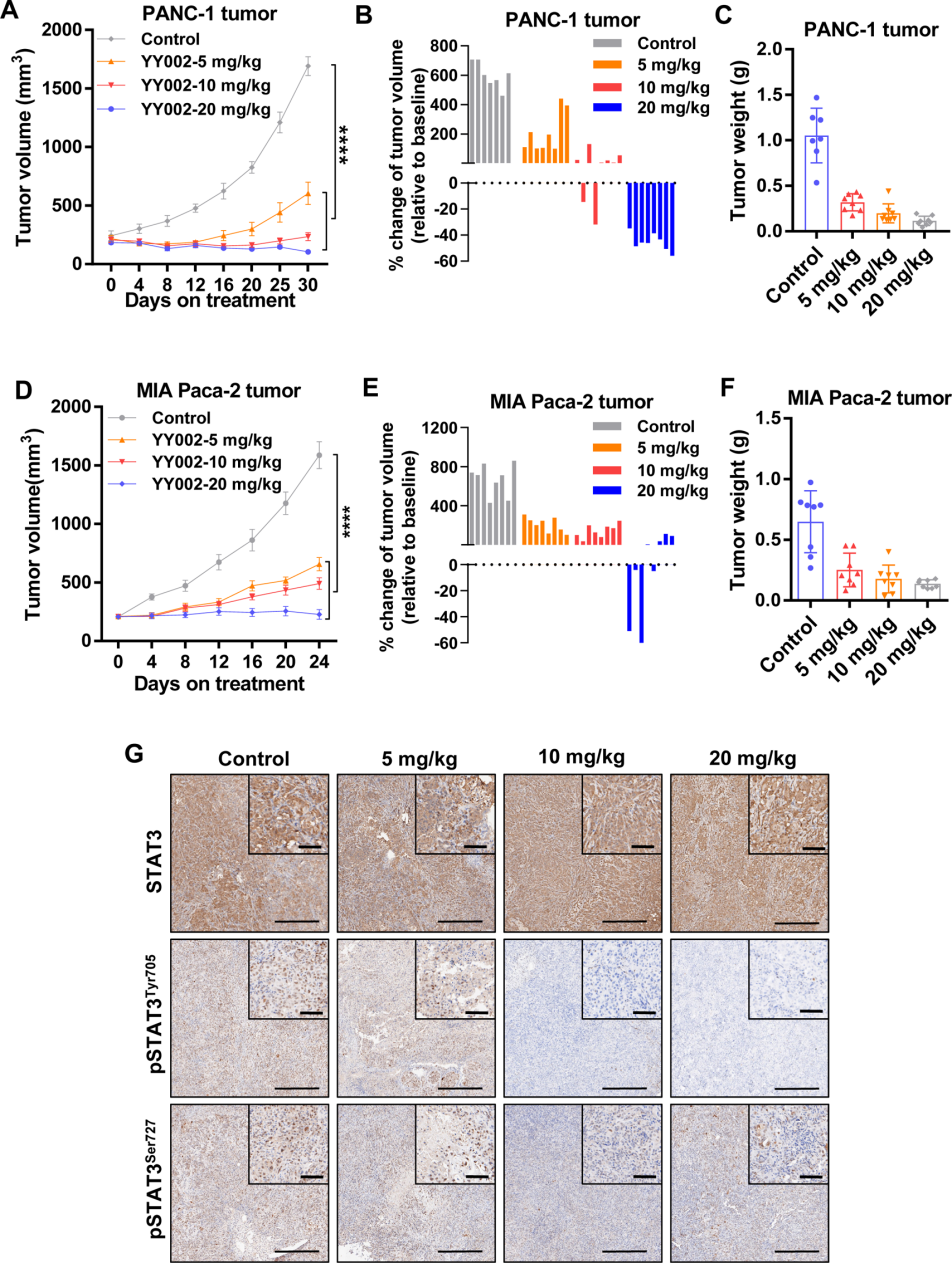
YY002 inhibits pancreatic cancer growth *in vivo*. (A, B) The PANC-1 tumor volumes of mice were recorded every 4–5 days (*n* = 8). Data shown as mean ± SEM * *P* < 0.05, * * *P* < 0.01 and **** *P* < 0.0001 by One-way ANOVA followed multiple comparison. (C) At the end of the experiment, the tumors in each group were excised, weighed and counted. (D-E) MIA PaCa-2 cells were injected into mice which were treated with different concentrations of YY002 orally (*n* = 8). (F) The tumor volume was recorded every 4 days and after 24 days of administration, the tumors were harvested. Data shown as mean ± SEM * *P* < 0.05, * * *P* < 0.01 and **** *P* < 0.0001 by One-way ANOVA followed multiple comparison. At the end of the experiment, the tumors in each group were excised, weighed and counted. (G) Quantification of STAT3, pSTAT3^Tyr705^, and pSTAT3^Ser727^ immuno-staining and representative images of PANC-1 tumor.

To evaluate the antitumor growth activities of YY002 against pancreatic cancer *in vivo*, we established an animal model of pancreatic cancer using PANC-1, and set the dose of YY002 from 5 mg/kg/d to 20 mg/kg/d, which was administered orally. As the results show, both the tumor volume (Figure 5A,B) and tumor weight (Figure 5C) were significantly inhibited after YY002 treatment. At the dose of 20 mg/kg, YY002 caused a significant potent tumor regression *in vivo*, and the tumor growth inhibition (TGI) rate was 93.56 ± 1.58%. In another mouse xenograft model, similar results were observed. YY002 suppressed MIA PaCa-2 tumor growth in a concentration-dependent manner (Figure 5D–F). The TGI rates of each dose were 61.24 ± 9.68% (20 mg/kg), 70.91 ± 7.84% (10 mg/kg), and 85.06 ± 9.28% (5 mg/kg). FLLL32 was a represented STAT3 inhibitor that suppressed the tumor growth of breast cancer, PDAC *in vitro* and *in vivo*.^32^ To examine the efficacy of YY002 and FLLL32 at inhibiting tumor growth i*n vivo,* we established an animal model of pancreatic cancer using BxPC3 cells. Both of YY002 or FLLL32 treatment caused a significant inhibition in tumor growth compared with vehicle treatment. Moreover, YY002 was orally available, and also achieved superior inhibition of tumor growth *in vivo* upon comparing with FLLL32 treatment groups (Supplementary Figure S6).

Of note, the effect of YY002 therapy was well tolerated, since no animals in any group exhibited systematic toxicity in these studies (Supplementary Figure 7A,B). The liver enzymes, such as alanine aminotransferase (ALT) and aspartate aminotransferase (AST), were detected in two groups of mice treated with vehicle and YY002, respectively. The data showed that YY002 did not induce hepatic toxicity after treatment with YY002 (Supplementary Figure 7C). Besides, there was also no significant difference in serum blood urea nitrogen (BUN) and creatinine (CREA) levels between vehicle and YY002 groups (Supplementary Figure 7C). The hematology analyses also revealed that YY002 treatment did not have a significantly influence in the counts of white blood cells, lymphocytes, monocytes and neutrophils versus control mice (Supplementary Figure 7D). Altogether, YY002 is well tolerated *in vivo.*

To further investigate whether YY002 inhibits STAT3 phosphorylation *in vivo*, we detected STAT3, pSTAT3 (Tyr705) and pSTAT3 (Ser727) in PACN-1 tumors. Pharmacodynamic (PD) analysis demonstrated that YY002 did not suppress STAT3 expression *in vivo* (Figure 5G, Supplementary Figure S7E). Besides, a p.o. dose of YY002 at 5 mg/kg to 20 mg/kg significantly suppressed the phosphorylation of STAT3 at Tyr705 and Ser727 in PDAC tumors (Figure 5G, Supplementary Figure S7F,G). These effects were consistent with the results of YY002 on pancreatic cancer cell lines. Taken together, these data suggest that YY002 results in strong growth inhibitory activities in PDAC tumor models, and our PD analysis demonstrated that YY002 simultaneously suppressed the phosphorylation of STAT3 at Tyr705 and Ser727 *in vivo*.

### YY002 Inhibits Pancreatic Cancer Metastasis

**Figure 6:**
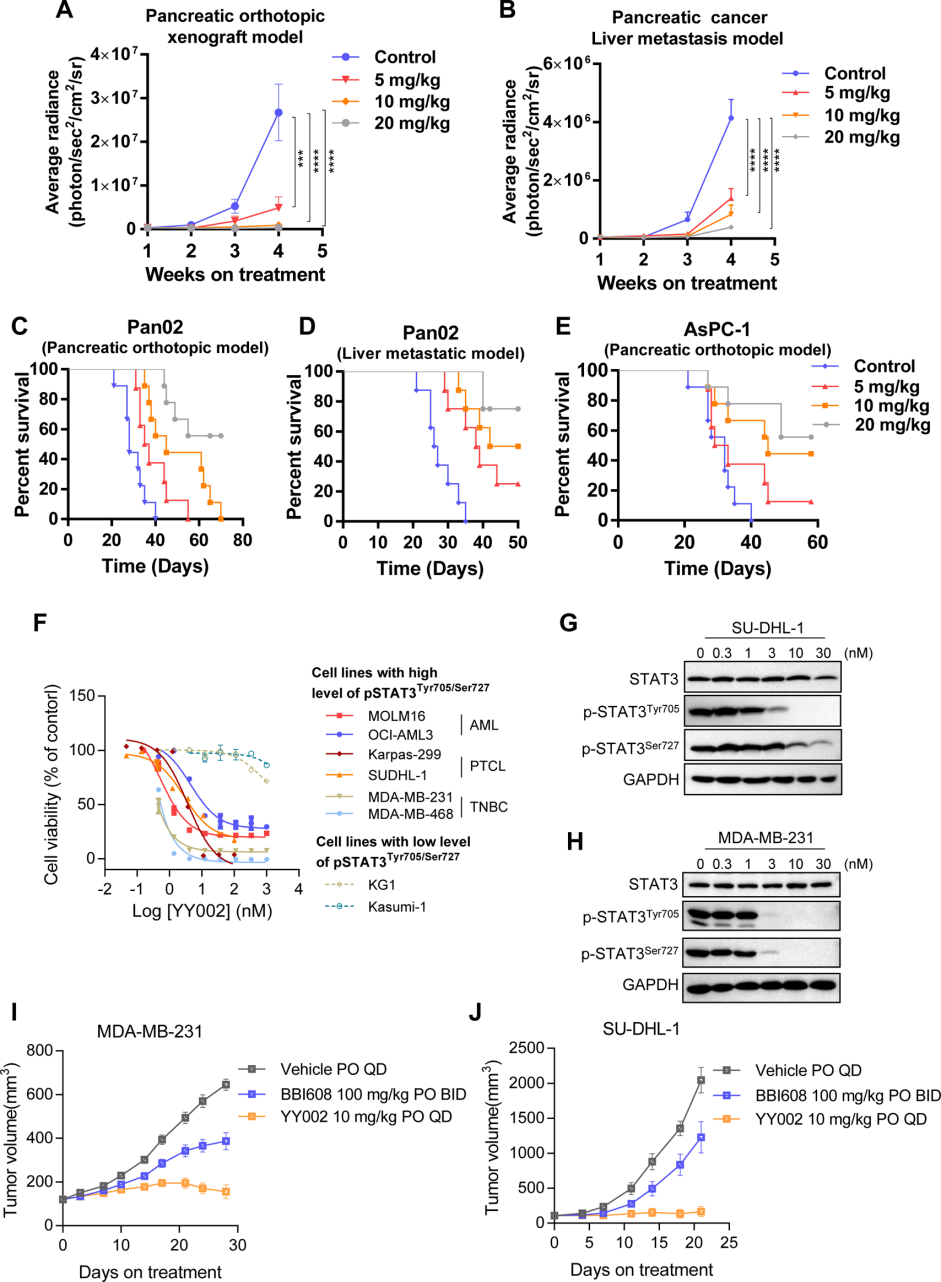
YY002 inhibits metastasis of pancreatic cancer *in vivo*. (A) PAN02-Luciferase cells were implanted orthotopically into the pancreas tails of male C57/BL6 mice. Different concentrations of YY002 were given orally for 4 weeks. Quantification of bioluminescence in pancreatic orthotopic xenograft model(*n* = 7). (B) PAN02-Luciferase cells were inoculated intravenously into male C57/BL6 mice. Quantification of bioluminescence in pancreatic cancer liver metastasis mouse model (*n* = 8). (C) Overall survival rates of the additional independent orthotopic of pancreatic cancer. Log-rank (mantel-cox) test was used (*n* = 9 in Control, 10 mg/kg, 20 mg/kg groups; *n* = 8 in 5 mg/kg group). (D) Overall survival rates of the additional independent liver metastatic models of pancreatic cancer. Log-rank (mantel-cox) test was used (*n* = 8 in each group). (E) AsPC-1 cells were implanted orthotopically into the pancreas tails of mice, and then mice were treated with different concentrations of YY002 for 4 weeks, The survival time was counted continuously (*n* = 9 in Control, 10 mg/kg, 20 mg/kg groups; *n* = 8 in 5 mg/kg group). Data shown as mean ± sd. * *P* < 0.05, ** *P* < 0.01, *** *P* < 0.001 and **** *P* < 0.0001 by One-way ANOVA followed multiple comparison. (F-J). YY002 induced the death of other STAT3-dependent tumors. (F) YY002 induced various STAT3-dependent cancer cells death. The tumor cells treated with YY002 in different concentrations, the cell viabilities were determined by MTS assays (*n* = 2). (G, H) The STAT3 phosphorylation and the expression of STAT3 were measured by Western Blot in MDA-MB-231 and SUDHL-1 cells after treatment with YY002. (I) MDA-MB-231 cells were injected into mice which were treated with YY002 or BBI608 orally (*n* = 10). (J) SUDHL-1 cells were injected into mice which were treated with YY002 or BBI608 orally (*n* = 10).

Owning to the potent antitumor growth effects *in vivo*, and recent reports suggesting that STAT3 is involved in key steps of pancreatic cancer metastasis,^33,34^ we next tested the effects of YY002 administration on pancreatic tumors *in vivo* using an orthotopic pancreatic cancer mouse model and a liver metastatic mouse model. In the orthotopic pancreatic cancer mouse model constructed by PAN02, YY002 inhibited tumor growth and metastasis in a concentration-dependent manner (Figure 6A). Since YY002 markedly inhibited tumor growth and metastasis in an orthotopic pancreatic cancer mouse model, we therefore carried out further experiments to confirm whether YY002 inhibited liver metastasis of pancreatic tumors. Advanced pancreatic cancer frequently carries fatal liver metastases with no effective treatment available. By injecting PAN02 cells into the mouse spleen, the tumor directed metastasis to the liver. And YY002 significantly inhibited tumor signal (Figure 6B). We hypothesized that YY002-mediated suppression of metastasis of pancreatic cancer would prolong survival of tumor-bearing mice. To assess this, we determined survival rates of mice that received vehicle or drug treatments in the independent pancreatic mouse metastatic models. YY002 significantly prolonged the survival of the tumor-bearing mice that constructed by PAN02 cells (Figure 6C,D). Besides, YY002 also significantly increased overall survival in the orthotopic model constructed from another highly metastatic human pancreatic cancer cell line AsPC-1, and 60% of mice in the high concentration group survived (Figure 6E). In conclusion, we demonstrated that YY002 strongly inhibited pancreatic tumor metastasis *in vivo* and significantly prolonged the survival of mice with pancreatic tumor metastasis in a preclinical pancreatic cancer mouse model.

### YY002 Induced the Death of Other STAT3-Dependent Tumors *in Vitro* and *in Vivo*

Considering the critical role of STAT3 for the tumor progression, targeted STAT3 signaling represents a promising therapeutic approach for several tumors, including triple negative breast cancer (TNBC),^35,36^ acute myeloid leukemia (AML),^37^ and peripheral T-cell lymphoma (PTCL).^38^ We then selected six non-PDAC cell lines (MOLM16, OCI-AML3 AML cell lines, Karpas-299, SUDHL-1 PTCL cell lines; MDA-MB-231, MDA-MB-468 TNBC cell lines) representing other human tumor types that highly depended on STAT3. As results shown, YY002 treatment led to the specific death of STAT3-dependent cell lines derived from various tumor types (Figure 6F). Moreover, YY002 potently inhibited STAT3 Tyr705 and Ser727 phosphorylation in TNBC cells (MDA-MB-231) and PTCL cells (SUDHL-1) (Figure 6G,H). Importantly, our study demonstrates YY002′s potent antitumor efficacy *in vivo*, notably causing significant regression in STAT3-dependent tumors (Figure 6I,J).

There are several ongoing clinical stage STAT3 inhibitors currently being investigated in advanced tumor patients. For instance, Napabucasin (BBI608) is an orally available STAT3 and cancer cell stemness inhibitor,^39^ which has finished phase III studies in patients with advanced tumor in combination with standard chemotherapies.^40^ In TNBC and PTCL models, oral administration of YY002 demonstrated superior antitumor efficacy compared to BBI608. These findings underscore the immense therapeutic potential of YY002 for treating STAT3-dependent tumors (Figure 6I,J).

## Discussion

STAT3 is a member of the STAT transcription factor family, which promotes tumor progression by regulating the expression of genes related to cell proliferation, survival, metastasis, and immune evasion.^41^ STAT3 is abnormally constitutively activated in various tumors,^42−47^ such as pancreatic cancer, liver cancer, gastric cancer, and acute myeloid leukemia, which contributes to the poor prognosis of these patients. Thus, STAT3 is a promising drug target for cancer treatment. However, despite over 20 years of effort, the development of STAT3 inhibitors has proven to be particularly challenging. One major challenge is the difficulty of acquiring highly selective STAT3 inhibitors that discriminate between the highly structurally homologous STAT family members. The other major challenge is that phosphorylation of STAT3 at Tyr705 along with Ser727 is required for full activation of STAT3; most reported STAT3 inhibitors antagonize STAT3-Tyr705 phosphorylation while ignoring the contribution of Ser727 phosphorylation. Herein, we present a highly potent and selective small-molecule STAT3 inhibitor, and validated its on-target efficacy using the following approaches. First, we confirmed that the compound inhibited STAT3 Tyr705 and Ser727 phosphorylation and the corresponding transcriptional activities and mitochondrial OXPHOS. Second, the compound selectively bound STAT3 over other STAT members. The binding domain was located in the SH2 domain, and binding residues and a binding model was also further clarified by computational modeling and direct binding assays. Third, we characterized the selective killing profile of this small-molecule STAT3 inhibitor by testing the anticancer activities of STAT3-negative PC3 cells and a variety of STAT3-dependent tumor cells, including PDAC cells, and liver cancer cells. YY002 activity depended on STAT3, as confirmed by our cell viability assay using stable shNC and shSTAT3 cells. Taken together, this study presented a novel small-molecule STAT3 inhibitor, which is highly selective for STAT3, and able to potently suppress STAT3 Tyr705 and Ser727 phosphorylation *in vitro* and *in vivo*.

Our study has a number of important implications. First, this study may provide a basis for the evaluation of STAT3 inhibitors in PDAC treatment. As numerous researchers reported, STAT3 is persistently activated in pancreatic cancer and is associated with poor prognosis of disease.^42,48,49^ Inhibition of STAT3 expression or blocking its activities by signaling pathway inhibitors suppresses tumor growth and metastasis.^50,51^ Conditional knockdown of STAT3 in the mouse pancreas had no significant effect on the pancreas development and function,^52^ demonstrating STAT3 is a favorable safety target for pancreatic cancer. Our results also further clarify that inhibition of Ser727 phosphorylation by YY002 induced mitochondrial dysfunction, leading to metabolic synthetic lethality in cancer cells. Since it induces a significant inhibition of OXPHOS, YY002 is expected to be a promising therapeutic strategy for OXPHOS-addicted tumors. In a subpopulation of brain tumors, due to the mutation or deletion of glycolysis-related genes such as ENO1,^49,53^ the tumors are glycolysis-deficient, thus heavily dependent on OXPHOS. Meanwhile, SWF/SNF mutations, which frequently occur in pancreatic cancer, melanoma, NSCLC, and other tumors,^15,54−56^ induce a targetable dependence on OXPHOS. Thus, through the development and characterization of YY002, we now provide an alternative therapeutic approach for OXPHOS-additive tumors.

Indeed, our work still has several limitations needing further exploration. For instance, the effect of YY002 on the tumor microenvironment merits further studies. Accumulating evidence suggests that STAT3 Tyr705 phosphorylation regulates the tumor microenvironment by enhancing immune evasion, cross-talk between tumor cells and cancer-associated fibroblasts (CAFs), and the remodeling of stromal cells.^1,57^ Also, the role of Ser727 phosphorylation in regulation of the tumor microenvironment remains poor understood. Hence, we will further explore the impact of YY002 on the tumor microenvironment, and also investigate the distinctive role of STAT3 Tyr705 or Ser727 phosphorylation in modulating the tumor microenvironment. Additionally, we have confirmed that YY002 exhibits specific binding to the STAT3 SH2 domain, identifying its potential binding amino acids. Importantly, these amino acids might play a pivotal role in STAT3′s functional modulation (Supplementary Figure S2D). Consequently, in our subsequent research, we will persist in optimizing and modifying the compound to effectively target these crucial regions. Upon YY002 binding with STAT3, a noticeable inhibition of STAT3′s dual phosphorylation was observed. We speculate whether this mechanism aligns with that of other STAT3 inhibitors. Studies have been revealed that several compounds bind to STAT3, inducing the inhibition of dual STAT3 phosphorylation^58−61^ Notably, some compounds can solely inhibit the STAT3 Ser727 phosphorylation.^62^ Furthermore, we have ruled out the potential impact of YY002 on upstream kinases, as our results indicate that YY002 does not significantly affect the activity of upstream kinases of STAT3 (Figure 2F). Therefore, our hypothesis suggests that YY002 may not directly inhibit kinase activity. Instead, after binding to STAT3, YY002 could potentially influence the interactions between STAT3 and kinases related to Tyr705 (e.g., JAK, EGFR) and Ser727 phosphorylation (e.g., ERK1, JNK1), ultimately leading to a reduction in STAT3 phosphorylation. To validate these hypotheses, additional comprehensive investigations are warranted in future studies.

In summary, we present a novel small-molecule STAT3 inhibitor YY002, which simultaneously inhibited STAT3 Tyr705 and Ser727 phosphorylation. YY002 potently inhibited pancreatic tumor growth and metastasis, and also exhibited favorable pharmacokinetics and an acceptable safety profile. Therefore, this study not only evaluates the potential value of the YY002 for the treatment of PDAC tumors, but also provides new insights to develop the novel STAT3 inhibitors targeting STAT3 Tyr705 and Ser727 phosphorylation.

## Data Availability

All the data supporting the findings of this study are available from the corresponding author on reasonable request.

## Supporting Information Available

Experimental procedures for the synthesis of YY002; additional materials and methods; and Figures S1–S7, Tables S1–S3 (PDF)
