# Supplementary material for: Discovery of the Highly Selective and Potent STAT3 Inhibitor for Pancreatic Cancer Treatment
Source: ACS Cent Sci. 2024 Feb 10;10(3):579–94. doi: 10.1021/acscentsci.3c01440 (PMC10979493; doi:10.1021/acscentsci.3c01440)
Supplement: Supplementary file 1 — oc3c01440_si_001.pdf [file oc3c01440_si_001.pdf]

Supporting information

Discovery of the highly selective and potent STAT3 inhibitor for pancreatic cancer treatment

Huang Chen[1, 2#], Aiwu Bian[1, 2#], Wenbo Zhou[1, 2#], Ying Miao[1#], Jiangnan Ye[1], Jiahui Li[3], Peng He[1], Qiansen Zhang[1], Yue Sun[1], Zhenliang Sun[3], Chaowen Ti[1], Yihua Chen[1*], Zhengfang Yi[1*], Mingyao Liu[1, 2*]

[1] Shanghai Key Laboratory of Regulatory Biology, Institute of Biomedical Sciences and School of Life Sciences, East China Normal University, Shanghai 200241, P.R. China

[2] Shanghai Yuyao Biotech Co., LTD. Shanghai, 200241, China;

[3] Southern Medical University Affiliated Fengxian Hospital, 201499, Shanghai, China

# These authors contributed equally to this work.

Correspondence to: Zhengfang Yi, Ph.D., Mingyao Liu, Ph.D., or Yihua Chen, Ph.D., Institute of Biomedical Sciences, East China Normal University, 500 Dongchuan Rd, Shanghai 200241, China (e-mail: zfyi@bio.ecnu.edu.cn, myliu@bio.ecnu.edu.cn, yhchen@bio.ecnu.edu.cn. Phone: 86-21-54345016; Fax: 86-21-54344922).

Table of Contents

Supplementary Methods and Materials;..S3

Supplementary Figures..S7

Supplementary Tables..S14

Supplementary Methods and Materials;

Chemicals

YY002 was synthesized in our laboratory, the detailed information is described in the patent[PCT/CN2020/102461].(1-methyl-6-((5-(3-(4-(trifluoromethyl)phenyl)-1,2,4-oxadiazol-5-yl)pyrazin-2-yl)oxy)-1H-indol-2-yl)(4-(4-(2,2,2-trifluoroethoxy)benzyl)piperazin-1-yl)methanone (YY002) ^{1}H NMR (500 MHz, $CDCl_3$) δ 9.02 (s, 1H), 8.65 (s, 1H), 8.34 (d, J = 8.1 Hz, 2H), 7.78 (d, J = 8.2 Hz, 2H), 7.68 (d, J = 8.6 Hz, 1H), 7.30 (d, J = 7.5 Hz, 2H), 7.22 (s, 1H), 6.98 (dd, J = 8.5, 1.8 Hz, 1H), 6.92 (d, J = 8.4 Hz, 2H), 6.63 (s, 1H), 4.35 (q, J = 8.1 Hz, 2H), 3.85-3.75 (m, 7H), 3.53-3.52 (m, 2H), 2.51-2.50 (m, 4H).^{13}C NMR (125 MHz, $CDCl_3$) δ 173.21, 168.36, 162.67, 162.31, 156.91, 149.07, 142.92, 138.25, 136.49, 133.28, 130.65, 129.92, 128.19, 126.07, 126.04, 124.95, 124.82, 122.92, 122.79, 122.36, 115.02, 114.64, 104.00, 102.73, 77.16, 66.22, 65.94, 62.19, 31.52, 31.06. HRMS (ESI) for C36H30F6N7O4 $[M + H]^+$: calcd738.2258,found738.2259.(1-methyl-6-((5-(3-(2-(trifluoromethyl)phenyl)-1,2,4-oxadiazol-5-yl)pyrazin-2-yl)oxy)-1H-indol-2-yl)(4-(4-(2,2,2-trifluoroethoxy)benzyl)piperazin-1-yl)methanone.

The compounds including Stattic, BP-1-102, BBI-608, AZD1480 and C188-9 were purchased from Selleck.

Cell Lines, Culture and Reagents

Human pancreatic cancer cell lines (Capan-2, PANC-1, MIA PaCa-2, CFPAC-1, SW1990, AsPC-1, BxPC-3 and HPAC), human prostate cancer cell line (PC3), human

acute myeloid leukemia cell line (KG1), human atrial fibroblast (HAF), human umbilical vein endothelial cell line (HUVEC), human embryonic kidney cell line (HEK293T) was purchased from Cell Bank/Stem Cell Bank of the Chinese Academy of Sciences and American Type Culture Collection (ATCC). Capan-2, PANC-1 and HEK293T were cultured in Dulbecco's Modified Eagle's Medium (DMEM) supplemented with 10% fetal bovine serum (FBS) and 1% penicillin-streptomycin, and additional 2.5% horse serum was added in the medium of MIA PaCa-2; AsPC-1, BxPC-3, PC3 and KG1 were cultured in RPMI 1640 medium supplemented with 10%-20% FBS and 1% penicillin-streptomycin; HPAC was cultured in Dulbecco's Modified Eagle Medium/Nutrient Mixture F-12 medium (DMEM/F-12), SW1990 was cultured in Leibovitz's L-15 Medium, CFPAC-1 was cultured in Iscove's Modified Dulbecco's Medium. HUVEC was cultured in Ham's F-12K supplemented with 0.1mg/mL Heparin, 0.03-0.05mg/mL ECGs, 10% FBS and 1% penicillin-streptomycin. HAF was cultured in Fibroblast Medium supplemented with 10% FBS, 1% Fibroblast Growth Supplement (FGS) and 1% penicillin-streptomycin.

Cell viability

Cell viability was measured by MTS assay following the manufacturer's instructions. Briefly, cancer cells or normal cells were seeded in 96-well plates at appropriate densities (5000-20000 per well in 96 plates) overnight, followed by 72 h of YY002 treatment. Aqueous One solution (MTS) was then added. The OD values at 490 nm were acquired.

Luciferase reporter assay

The HEK293T cells were seeded in 24-well plates, The 500 ng STAT3 luciferase plasmids (pGL4.47 luc2p/SIE/Hygro) vector (E4041, Promega), 500 ng STAT3-WT or mutation plasmids and 10 ng Renilla plasmids were co-transfection into each well of plates with Lipofectamine 2000 reagent (Invitrogen) and incubated for 20-24 h. Then, the cells were added with IL-6 (20 ng/mL). Luciferase assays were performed by using the Dual Luciferase Reporter Assay System (Promega) according to manufacturer's instructions. The firefly luciferase activities were normalized against Renilla luciferase activities.

Mitochondrial membrane potential assay

Mitochondrial membrane potential was assessed following the manufacturer's instructions (Beyotime, C2006). Briefly, the cells were seeded in 6-well plates, YY002 treated for 24h. Then the cells were washed with PBS and incubated with JC－1 probe in 1× dye working buffer for 20 min at 37° C. After washing with cleaning buffer, JC－1 monomers and JC－1 aggregates were detected with Flow cytometry to indicate mitochondrial membrane potential.

Reactive Oxygen Species (ROS) Assay

ROS assay was performed following the manufacturer's instructions (Beyotime, S0033S). Briefly, the pancreatic cancer cells were seeded in 6-well plates, YY002 treated for 20h. Then the cells were wash with cold PBS for three times. Dilute 2',7'-Dichlorodihydrofluorescein diacetate (DCFH-DA) with serum-free culture medium at a ratio of 1:1000 to achieve a final concentration of 10 μM. Remove the cell culture

medium, and add 1mL of the diluted DCFH-DA. Incubate at 37°C for 20 minutes, and wash the cells three times with serum-free cell culture medium to thoroughly remove DCFH-DA that has not entered the cells. ROS signal was detected by FACS.

Hematoxylin-eosin (H&E)

For the HE staining, all of the samples were immediately fixed in 4% neutral buffered formaldehyde for 24 hours, progressively dehydrated in solutions containing an increasing percentage of ethanol (75, 85, 95 and 100%, v/v) and then embedded into paraffin. 4 μm sections were cut from the paraffin blocks. H&E samples were stained with hematoxylin and eosin to indicate nucleus and cytoplasm, respectively. Photos were taken by Leica photomicroscope.

Supplementary Figures

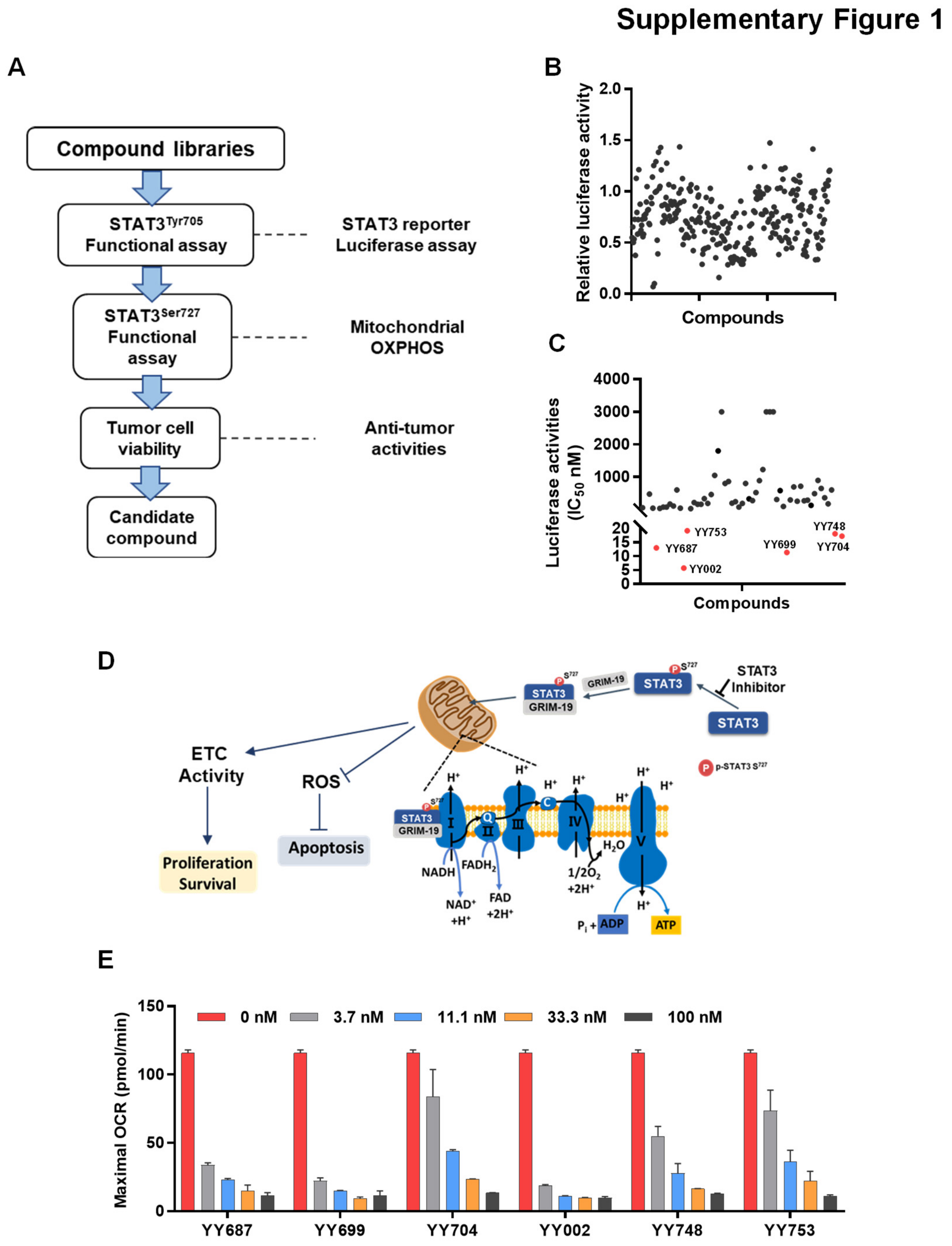

Supplementary Figure 1. Screening for novel STAT3 inhibitors that suppress Tyr705 and Ser727 phosphorylation. (A) Screening strategies to identify small molecule STAT3 inhibitors that suppressed STAT3 Tyr705 and Ser727 phosphorylation. (B) Preliminary screening results of STAT3 transcriptional assays. The concentration of tested compounds was 10 μM (n = 2). (C) The IC_{50} values of the selected compounds against STAT3 luciferase activities. (D) Diagram of STAT3 regulation of mitochondrial OXPHOS. (E) The selected compounds inhibited mitochondrial OXPHOS.

Supplementary Figure 2

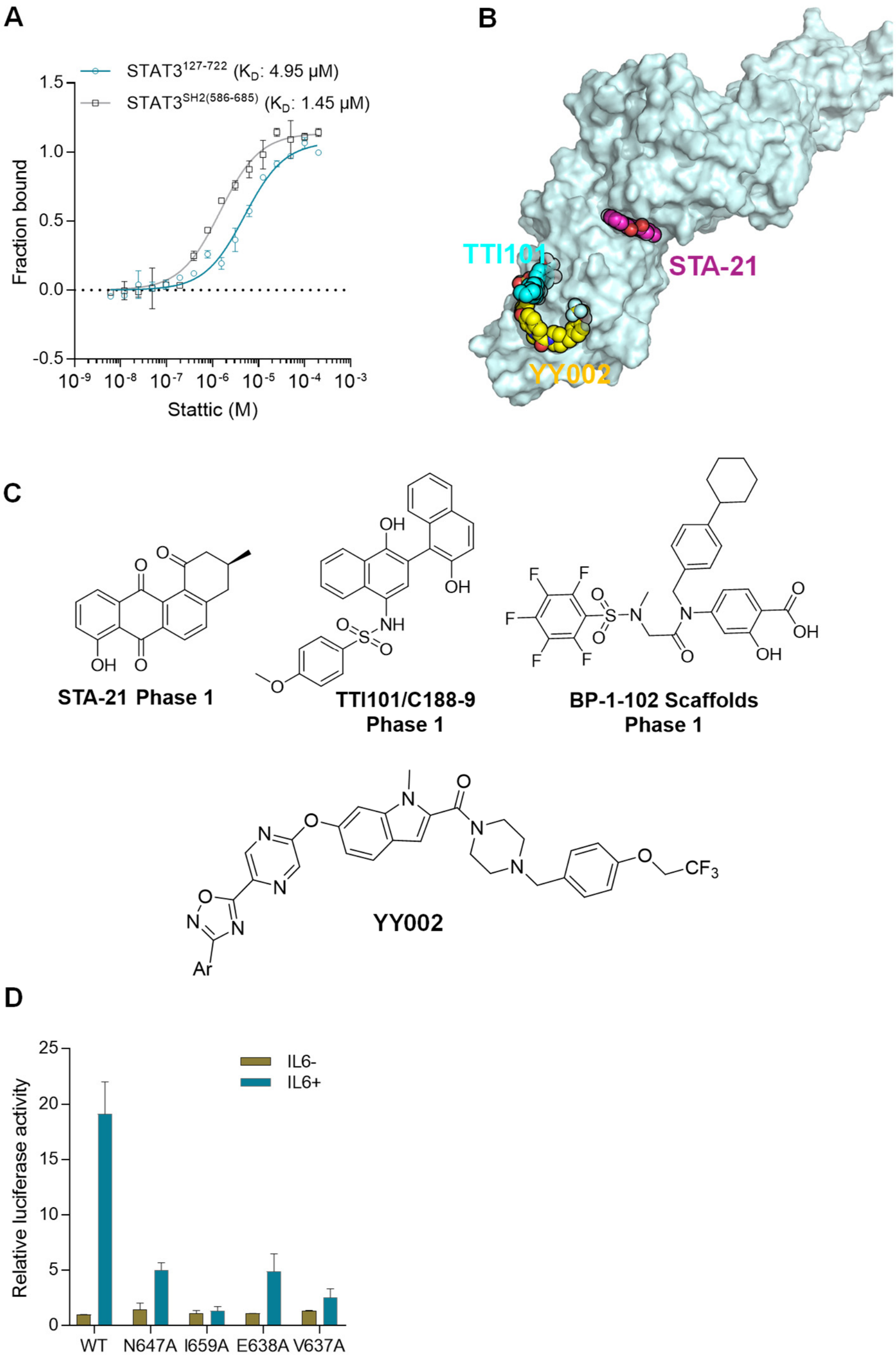

Supplementary Figure 2. (A) The binding affinities of Stattic and STAT3 protein (STAT3$^{127\text{-}722}$, STAT3$^{SH2\ (586\text{-}685)}$) were detected by MST experiments (n = 2). (B) Binding model of YY002 and the reported STAT3 inhibitors. (C) Chemical structure of YY002 and the reported STAT3 inhibitors. (D) The mutation in STAT3 SH2 domain directly inhibited STAT3transcriptional activities. STAT3 luciferase reporter assay was performed in HEK293T. Indicated STAT3 expression plasmids including WT, and SH2-domain point mutations were co-transfected with STAT3-luciferase reporter plasmids, and luciferase activities were measured. IL-6 (20 ng/mL) was used as an activator of STAT3 (n = 2).

Supplementary Figure 3

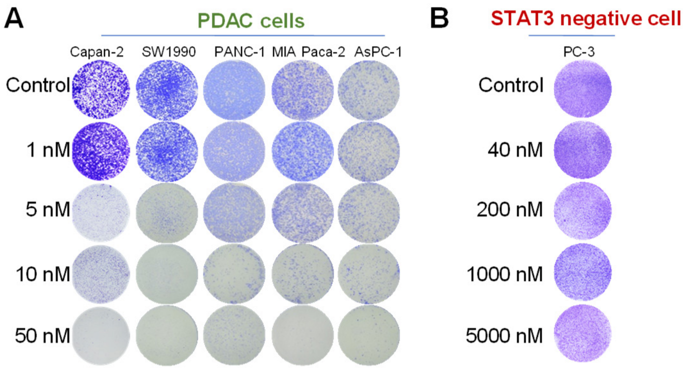

Supplementary Figure 3. YY002 selectively inhibits STAT3-dependent cell colony formation and STAT3 transcriptional activity. (A) YY002 inhibited PDAC cell colony formation. (B) YY002 did not inhibit STAT3 negative PC3 cell colony formation.

Supplementary Figure 4

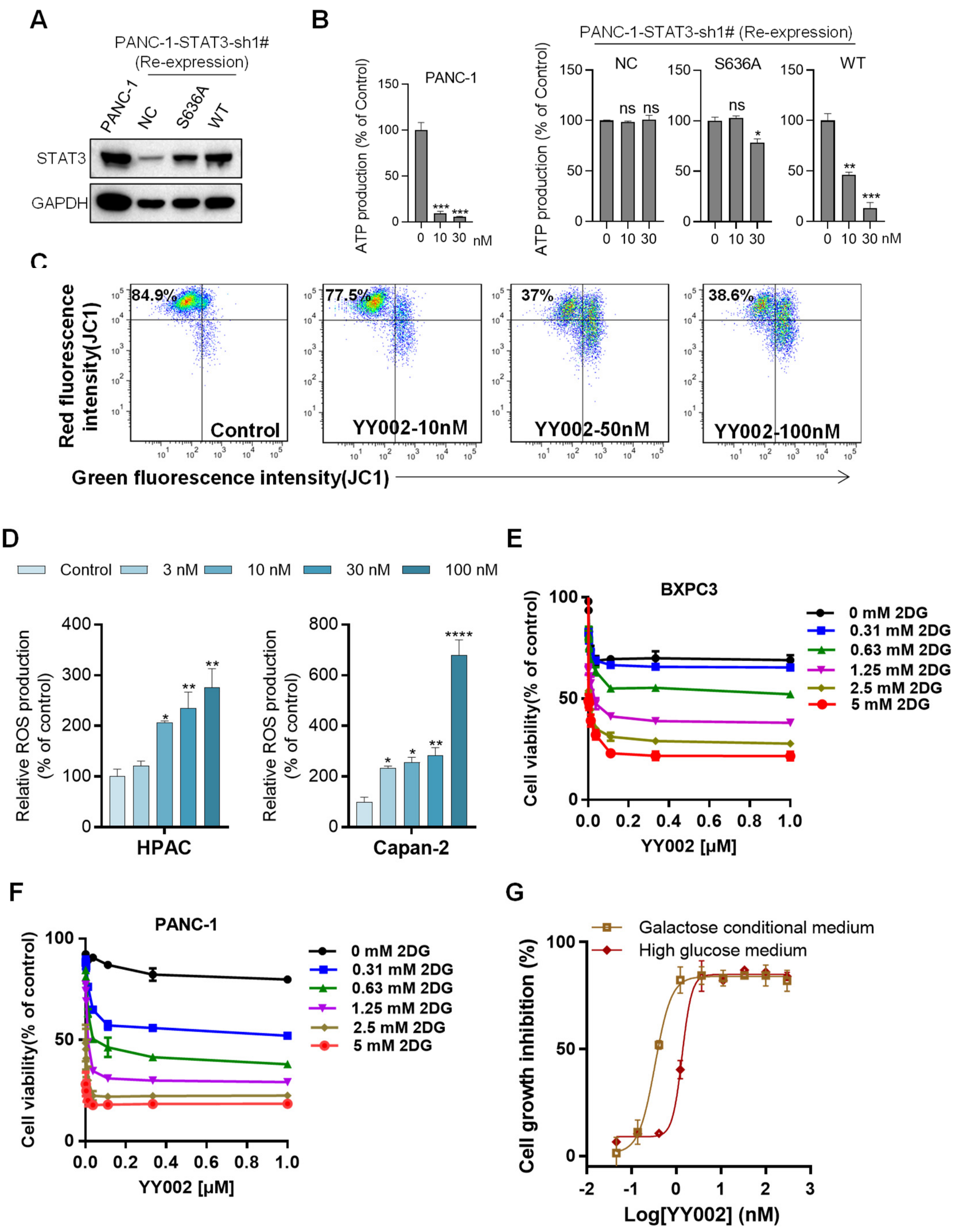

Supplementary Figure 4. YY002 impairs mitochondrial function. (A-B) The shSTAT3-1# PANC-1 cells that were infected with the indicated lentivirus expression vectors, and the transfection efficacy and ATP levels were detected (n=2). ns, P>0.5, ** P < 0.01 and *** P < 0.001 by One-way ANOVA followed multiple comparison. (C) Mitochondrial membrane potential (JC1 staining) in PANC-1 cells treated with YY002 at different concentrations for 2 h. (D) PDAC cells were treated with YY002 with indicated concentrations for 20h, and the ROS was detected (n = 2). (E-F) Proliferation of BxPC3 and PANC-1 cells treated with YY002 in the presence of 2-DG (n = 2). (G) The BxPC3 cells were replaced the medium to galactose conditional medium or high glucose medium, after 72h treatment of YY002, the cell viability was detected by MTS assay (n = 2).

Supplementary Figure 5

A

PK Parameters (Mice)		iv	po
Dose	mg·kg^{-1}	1.0	10
$t_{1/2}$	h	13.6	14.3
C_{max}	ng/mL	8154.4	4307.8
$AUC_{0\text{-}24}$	h*ng/mL	28492.8	89145.2
$AUC_{0\text{-}\infty}$	h*ng/mL	31926.9	102082.3
CL	mL/h/kg	31.3	98.0
$MRT_{0\text{-}t}$	h	12.7	15.0
Vd	mL/kg	616.0	2018.7
F(AUC0-t)	%	-	31.3

B

Species	Plasma $t_{1/2}$ (min)	Liver Microsomes $t_{1/2}$ (min)
Human	> 120	> 120
Dog	> 120	> 120
Monkey	> 120	> 120
Rat	> 120	> 120
Mouse	> 120	> 120

C

Protein	IC_{50} (μM)
hERG	> 25
CYP1A2	> 25
CYP2C9	> 25
CYP2C19	> 25
CYP2D6	> 25
CYP3A4-M	> 25

Supplementary Figure 5. YY002 showed adequate drug-like properties. (A) Pharmacokinetic parameters of YY002 following oral administration and intravenous injection in mice. **(B)** The plasma stability and metabolic stability in different species. **(C)** Summary of YY002 inhibition of key proteins for safety profiling.

Supplementary Figure 6

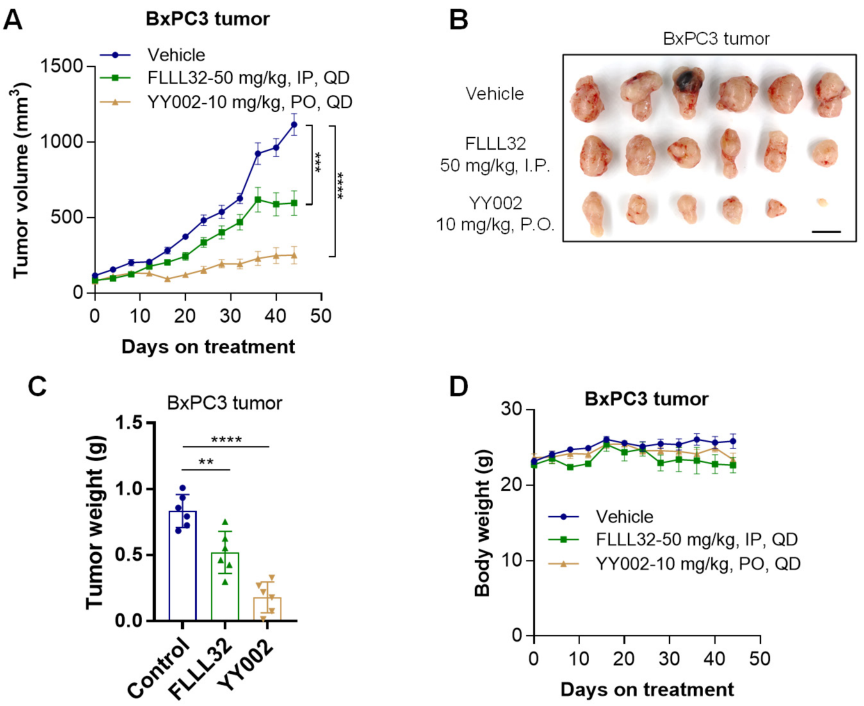

Supplementary Figure 6. YY002 and FLLL32 significantly inhibited tumor growth *in vivo*. (A) The BxPC3 tumor volumes of mice were recorded every 4 days (n = 6). Data shown as mean ± SEM. * $P < 0.05$, ** $P < 0.01$, *** $P < 0.001$ and **** $P < 0.0001$ by One-way ANOVA followed multiple comparison. (B-C) At the end of the experiment, the tumors in each group were excised, weighed and counted. Data shown as mean ± SEM. * $P < 0.05$, ** $P < 0.01$, *** $P < 0.001$ and **** $P < 0.0001$ by One-way ANOVA followed multiple comparison. Scale bar, 1cm. (D) The body weight of each group mice.

Supplementary Figure 7

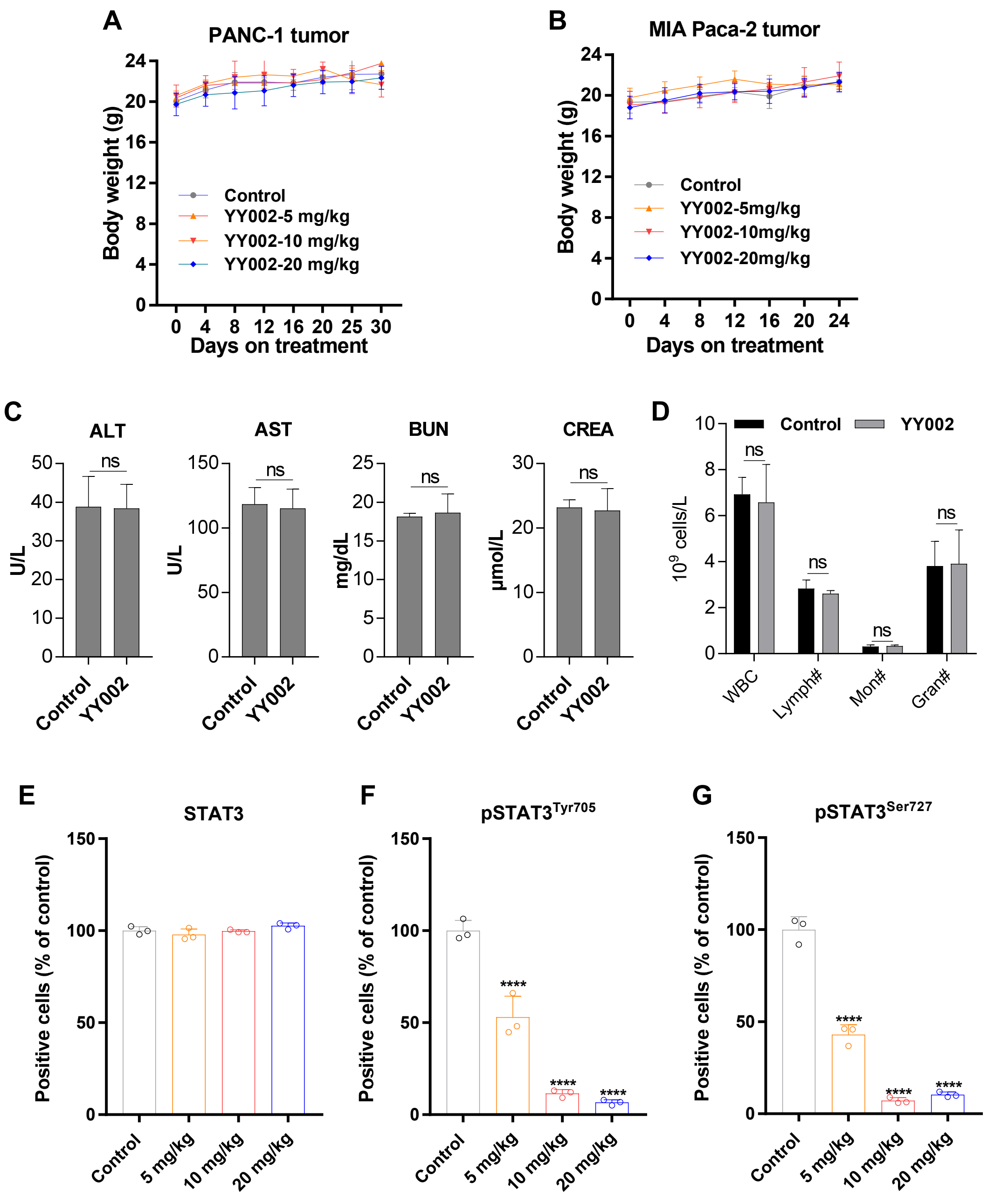

Supplementary Figure 7. YY002 is well-tolerated *in vivo*. (A) In the PANC-1 tumor model, the body weight of mice was recorded every 4-5 days (n =7 in control group, and n= 8 in YY002 treatment groups). (B) In the MIA Paca-2 tumor model, the body weight of mice was recorded every 4 days (n = 8 per groups). **(C)** The liver function tests were performed at post treatment to evaluate aminotransferase (ALT) and aspartate aminotransferase (AST) in different treatment groups. And the levels of serum creatinine (CREA) between vehicle and YY002 groups. YY002 (10 mg/kg, *P.O.*) **(D)** The number of different blood cell between vehicle and YY002 groups. White Blood Count (WBC), Lymphocyte number (Lymph#), monocytes number (Mon#), Neutrophil number (Gran#). YY002 (10 mg/kg, *P.O.*) (E-G) Quantification of STAT3 (E), pSTAT3^{Tyr705} (F), and pSTAT3^{Ser727} (G) immuno-staining of PANC-1 tumors. Data shown as mean ± sd. ns, P>0.5, **** P < 0.0001 by One-way ANOVA.

Supplementary Tables

Table S1. The sequence of shRNAs for silencing STAT3

Gene	Sequence (5’ – 3’)
shSTAT3-1#	GCACAATCTACGAAGAATC
shSTAT3-2#	GGCGTCCAGTTCACTACTA
shNC	CAACAAGATGAAGAGCACC

Table S2. List of antibodies used and their technical information

Primary Antibodies		
Name	Source	Catalog#
STAT3	CST	12640
p-STAT3^{Tyr705}	CST	9145
p-STAT3^{Ser727}	Abways	CY6500
GAPDH	Abcam	ab181602
c-Myc	Abcam	ab32072
Cyclin D1	Abcam	ab134175
Secondary antibodies		
Name	Source	Catalog#
Goat Anti-Rabbit IRDye 800CW	LI-COR	926-32211
Goat Anti-Mouse IRDye 800CW	LI-COR	926-32210

Table S3. The sequence of Real-time PCR primers

Gene	Forward primer (5’ – 3’)	Reverse primer (5’ – 3’)
β-actin	GTACGCCAACACAGTGCTG	CGTCATACTCCTGCTTGCTG
c-Myc	GTCAAGAGGCGAACACACAAC	TTGGACGGACAGGATGTATGC
Cyclin D1	GCTGCGAAGTGGAAACCATC	CCTCCTTCTGCACACATTTGAA
BCL-2	GGTGGGGTCATGTGTGTGG	CGGTTCAGGTACTCAGTCATCC
VEGF	CGAAACCATGAACTTTCTGC	CCTCAGTGGGCACACACTCC
